# A Predictive Model Has Identified Tick-Borne Encephalitis High-Risk Areas in Regions Where No Cases Were Reported Previously, Poland, 1999–2012

**DOI:** 10.3390/ijerph15040677

**Published:** 2018-04-04

**Authors:** Pawel Stefanoff, Barbara Rubikowska, Jakub Bratkowski, Zbigniew Ustrnul, Sophie O. Vanwambeke, Magdalena Rosinska

**Affiliations:** 1 Department of Epidemiology of Infectious Diseases and Surveillance, National Institute of Public Health—National Institute of Hygiene, 00-791 Warsaw, Poland; mrosinska@pzh.gov.pl; 2 Department of Population Health Monitoring and Analysis, National Institute of Public Health—National Institute of Hygiene, 00-791 Warsaw, Poland; brubikowska@pzh.gov.pl; 3 Institute of Environmental Protection—National Research Institute (IOS—PIB), 00-548 Warsaw, Poland; jakub.bratkowski@ios.edu.pl; 4 Department of Climatology, Jagiellonian University, 30-387 Krakow, Poland; zbigniew.ustrnul@uj.edu.pl; 5 Institute of Meteorology and Water Management, 30-215 Krakow, Poland; 6 Georges Lemaître Centre for Earth and Climate Research, Earth & Life Institute, Université Catholique de Louvain, 1348 Louvain-la-Neuve, Belgium; sophie.vanwambeke@uclouvain.be

**Keywords:** tick-borne encephalitis, ecologic study, epidemiologic determinants, land use predictors, zero-inflated Poisson model

## Abstract

During 1999–2012, 77% of the cases of tick-borne encephalitis (TBE) were recorded in two out of 16 Polish provinces. However, historical data, mostly from national serosurveys, suggest that the disease could be undetected in many areas. The aim of this study was to identify which routinely-measured meteorological, environmental, and socio-economic factors are associated to TBE human risk across Poland, with a particular focus on areas reporting few cases, but where serosurveys suggest higher incidence. We fitted a zero-inflated Poisson model using data on TBE incidence recorded in 108 NUTS-5 administrative units in high-risk areas over the period 1999–2012. Subsequently we applied the best fitting model to all Polish municipalities. Keeping the remaining variables constant, the predicted rate increased with the increase of air temperature over the previous 10–20 days, precipitation over the previous 20–30 days, in forestation, forest edge density, forest road density, and unemployment. The predicted rate decreased with increasing distance from forests. The map of predicted rates was consistent with the established risk areas. It predicted, however, high rates in provinces considered TBE-free. We recommend raising awareness among physicians working in the predicted high-risk areas and considering routine use of household animal surveys for risk mapping.

## 1. Introduction

Central European tick-borne encephalitis (TBE) is a viral disease characterized by a strongly focal distribution [1,2]. The virus persists in endemic areas by circulating in small mammals (mainly rodents) and is transmitted by an arthropod vector, commonly the tick *Ixodes ricinus* [1]. The TBE virus (TBEV) causes lifelong infection in ticks and can be transmitted transovarially, between mating ticks, and while co-feeding with other infected ticks on the same host. Humans and household animals are accidental tick hosts. Humans get infected predominantly through tick bites or, less commonly, by consumption of unpasteurized milk of infected sheep or goats [3]. TBEV circulation mainly depends on the abundance of tick vectors, and the abundance of competent rodent hosts [1,4,5]. Tick vector populations, in turn, rely on suitable conditions of humidity, soil and air temperatures, vegetation cover, and on the availability of large mammals. These factors were shown to affect TBEV transmission [6,7]. The complex interplay of multiple factors necessary for circulation of the virus may explain the strictly focal and limited occurrence of TBEV. Although various determinants of TBE endemicity have been extensively studied, specific aspects have often been investigated separately, for example the role of weather conditions [8], of land use [9,10], or of socio-economic determinants [11].

Moreover, many environmental factors, especially those related to wildlife, are not monitored systematically. Therefore, studies investigating TBE risk in a more comprehensive manner are usually limited to areas where detailed data are available or collected on purpose [10,12,13,14]. The need to distinguish between environmental factors, which define conditions for the natural life cycle of TBEV, and human behaviour, which affects the probability of human exposure to TBEV-infected ticks, has also been underlined [10].

**Figure 1:**
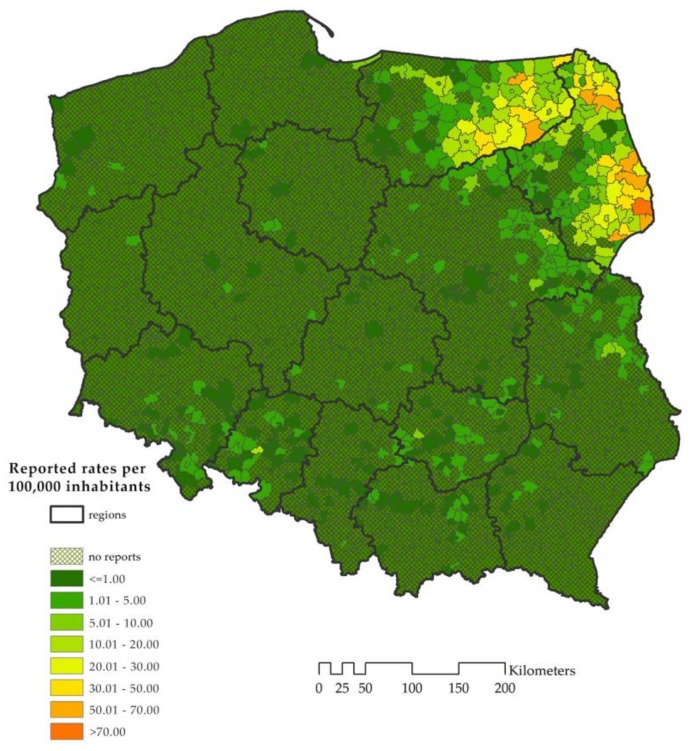
TBE reported rates by NUTS-5 administrative units, Poland, 1999–2012.

Only a limited number of Polish municipalities report TBE cases (Figure 1). These northeastern endemic areas border with the known and well documented foci in the Baltic states and, to a smaller degree, with highly active endemic areas in Czech Republic in the south.

**Figure 2:**
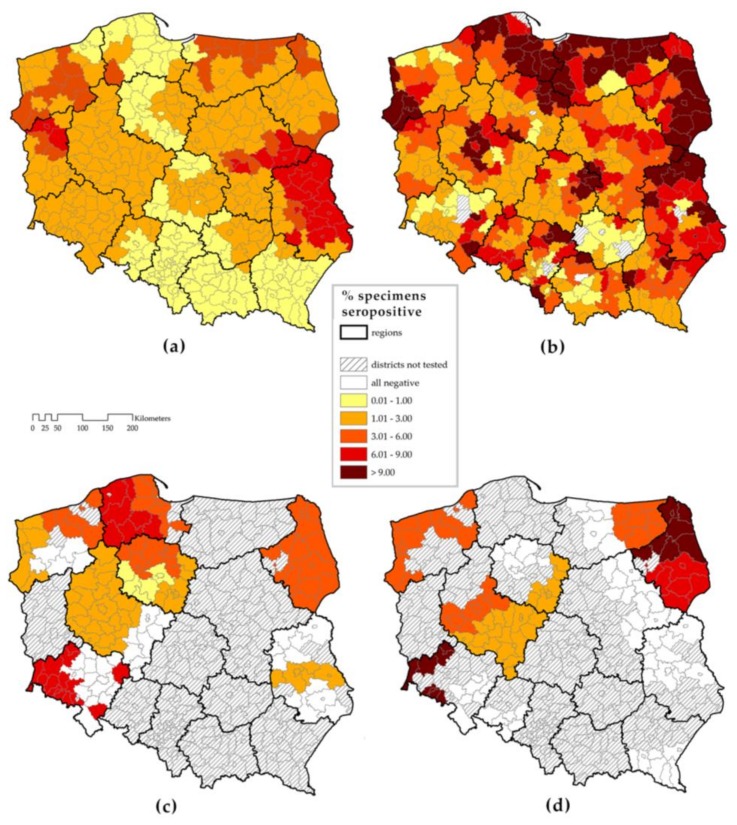
Previous studies of seroprevalence with national coverage at NUTS-4 district level: (**a**) in the years 1965–1967, 17,000 healthy subjects were selected from all districts in Poland, tested by complement fixation test (CFT) [15]; (**b**) in the years 1971–1972, 20,000 foresters were selected from most regions, tested by CFT [16]; (**c**) in the years 1996–2005, 1496 healthy subjects from selected provinces were tested by ELISA IgG [17]; and (**d**) in the years 2005–2007, 1122 goat sera from selected regions were tested by adapted ELISA IgG [18]. Note: the maps were redrawn based on original figures kept at the National Institute of Public Health, the owner of the data.

This limited geographical distribution may be, however, a surveillance artefact. Previous investigations have found comparable levels of seroprevalence in endemic and non-endemic areas (Figure 2) [15,16,17,18]. The presence of anti-TBEV antibodies might reflect travel to high-risk areas, vaccination (not in routine use in Poland), or exposure to contaminated milk coming from endemic regions. However, such levels of seroprevalence would only be expected in areas where people are regularly exposed locally to TBEV. In addition, the aetiology of meningoencephalitis is not routinely investigated by Polish physicians [19,20]. A survey in 2009 revealed that 70% of Polish hospitals do not have access to TBE serologic testing [19]. When physicians in 11 Polish provinces were offered free serologic diagnosis, the referral rate for testing was seven times lower in non-endemic, compared to the highly endemic areas [20]. This is a classic example when absence of evidence is incorrectly interpreted by stakeholders as evidence of absence of TBE risk.

Taking the assumption that most Polish regions do not record TBE cases, we aimed to identify which spatially-referenced meteorological, environmental, and socio-economic factors that are routinely measured best determine TBE human risk, in order to assess TBE risk across the Polish territory.

## 2. Materials and Methods

To build a comprehensive model to study the determinants of TBE endemicity, we reviewed all available data sources that give access to measurements at the lowest administrative level.

### 2.1. Spatial Resolution

The finest spatial resolution available in the routinely collected data is the municipality, NUTS level 5. Most of the municipalities in Poland have urban or rural status, but some are classified as mixed and contain distinct urban and rural components. The most detailed administrative division consists of four categories: urban and rural municipalities, urban parts of mixed municipalities, and rural parts of mixed municipalities. We decided to use only three categories and merged urban and rural parts of mixed municipalities. This was because some of the longitudinal data were not available for each of the four categories.

### 2.2. Study Period and Area

We decided to use data covering 14 years (1999–2012). In 1999, there was an administrative reform changing the administrative divisions of the country, making it difficult to build a coherent spatial dataset further back in the past.

We assigned the most temporally variable measurements to ‘dekads’—10-day periods routinely used in meteorology. Since each month was divided into three dekads, the third had variable length—from 8 to 11 days, depending on the duration of each month.

We selected TBE endemic areas as 17 (out of 379) NUTS-4 districts with a mean TBE incidence of over 5/100,000 during the study period, comprising 108 NUTS-5 municipalities. We assumed that TBE diagnosis was available in these endemic districts. Other districts were classified as of unknown endemicity due to concerns about diagnostic capacities in the area.

### 2.3. Selection of Variables for the Model

We prioritized data available at the NUTS-5 level that were routinely collected over the study period. This was to ensure that a working model could be routinely used in the future for predictive purposes. We reviewed the available data from the Central Statistical Office and from national agencies dealing with public health surveillance, forestry and environment. We also searched for map layers that would have sufficient resolution to assign measurements to NUTS-5 administrative units.

The definition of variables for the model is explained in Table 1 and in more detail in the Supplementary file 1. The TBE cases were assigned to their municipality of exposure, indicated in the case investigation form. Therefore, the case counts included both inhabitants of the respective municipalities and tourists visiting them. To address this, we adjusted the population denominator to the estimated tourist traffic. To assess the impact of adding non-residents on the model predictions we also performed sensitivity analyses including only cases among residents with the unadjusted population as denominator.

Meteorological measures were previously considered among the most important determinants of tick survival and activity [5,13,14], but also important factors of human outdoor activities [25]. They may play an important role in the predictive model as they are the most dynamically changing factors that are routinely measured and relatively easily accessible in public institutions. Among many measurements available, we have selected those routinely collected across the country, on a daily basis. We hypothesized that the air temperature affects both tick activity and influences outdoor activities of humans. Precipitation may be important for tick activity, as ticks can function only when air humidity is optimal. It has also a complex effect on human activity. Rainy days prevent people from spending time outdoors for recreation, but may also increase the likelihood that time is spent outdoors collecting mushrooms, an activity previously identified as a key determinant of individual-level TBE risk [26].

Land use factors were emphasized as key determinants of TBE risk, though likely the most temporally stable in our study. We prioritized variables related to forest accessibility: a combination of forested area, length of forest edge (a measure of forest fragmentation as introduced in [27]), average distance from forests, and forest road density, which permits better access to tick habitats by humans.

According to our previous investigations, socio-economic factors play a major role in TBE individual risk in Poland [26]. However, few were collected consistently at NUTS-5 level. We included unemployment, which was among the most important individual risk factors, established in the previous national case-control study.

### 2.4. Statistical Analysis

A total of 96.5% of the 54,432 observations (36 dekadal measurements × 108 municipalities × 14 years) had zero TBE case counts. We assumed that a zero-inflated Poisson model would fit the data better, which was confirmed by the Vuong test (*p*-value < 0.001). The mean and the variance of time series with TBE counts were similar (mean equal 0.040, variance equal 0.053), so we concluded that there was no overdispersion. We, therefore, decided to model the data as a panel (1 panel = 1 municipality) time series with zero-inflated Poisson regression (ZIP) that would accommodate both time series variables, as well as multiple categorical and continuous variables. The ZIP model is a combination of Poisson distribution with degenerated distribution concentrated on 0 (Equation (1)). The existence of a latent binary variable is assumed, that indicates from which of those two distributions each count comes from. Therefore, the ZIP model has two parts: a logistic part and a log-linear part [28]. Roughly the logistic part predicts whether or not the case count in a municipality during a dekad could be greater than zero and the Poisson part predicts the actual number of cases given that cases could occur:

(1) $$Y_{t}|u_{t}\sim Poisson\left(\left(1-u_{t}\right)\lambda_{t}\right),\;u_{t}\sim Bernoulli\left(\omega_{t}\right)$$

To select predictors in the ZIP model we firstly performed a time series analysis of the total number of TBE cases from all endemic municipalities by dekad. We used periodograms to determine cycles, checked the partial autocorrelation function and performed the Dickey-Fuller test for stationarity, which is necessary for building the predictive model as outlined above. Partial autocorrelation analysis allowed identification of the lag term to be included in the predictive model. An autocorrelation analysis was also performed for temperature and precipitation.

We considered focusing on temperature and precipitation measurements during the exposure period, which for TBE spans from two to four weeks before the onset of symptoms. In order to choose appropriate lags, we compared models including various combinations of lags using the Akaike Information Criterion (AIC). We examined only lags of order 1, 2, 3, and 4, which corresponds to taking into account observations from approximately 10, 20, 30, and 40 days, respectively.

We checked for linear associations between the logarithm of continuous variables and TBE incidence to assess the need for categorization.

Instead of fitting a final model by eliminating variables, we left all predictors in both, logistic and log-linear, parts of model. Since the interpretation of the ZIP model necessitates taking into account covariates from both parts of the model, its straightforward interpretation is not possible. Therefore, we used the marginsplot function (R software) to display predicted TBE rates, stratified by different levels of explanatory variables.

In order to investigate the goodness of fit of final ZIP model, we calculated the McFadden $\rho^{2}$ (Equation (2)):

(2) $$\rho^{2}=1-\frac{LL\left(\beta \right)}{LL\left(0\right)}$$

where LL(β) is the log-likelihood for the final model and LL(0) is the log-likelihood for the intercept-only model. The $\rho^{2}$ coefficient is a pseudo *R*^2^ measure for general linear models indicating the proportion of variability of data which is accounted for by the model.

Similarly, we calculated the $\rho^{2}$ coefficient for each risk factor separately, substituting the log-likelihood for the final model in Equation (2) by the log-likelihood of the model containing this particular risk factor only.

### 2.5. Mapping the Predicted Rates in All Municipalities

We applied the final model to predict the incidence rate in all municipalities in Poland, even those with no cases recorded. We summed the number of cases predicted for each dekad and calculated the annual predicted TBE incidences (1999–2012 averages) for each NUTS-5 municipality. Then we mapped the predicted rates on the NUTS-5 administrative division map, by assigning each rate to the centroid of the respective municipality polygon. Then we used the spline tension type interpolation, then visualized the rates for very small spatial units and their spatial variation more comfortably.

### 2.6. Validation of the Model

We assumed that the majority of Poland does not record TBE cases. Therefore, we could not validate the rates predicted by our model using the observed rates. We used the historical data from human seroprevalence surveys as the only valid source of information available both in the regions that report TBE and those non-reporting (Figure 2a–c). We calculated the Pearson correlation coefficients between the predicted rates by NUTS-4 areas and the proportion of their inhabitants tested positive in historical surveys. 

### 2.7. Statistical Software Used 

All statistical calculations were conducted in Stata^®^ 12 software (StataCorp LP, College Station, TX, USA). Figures were plotted using R 3.4.0. software (R Foundation for Statistical Computing, Vienna, Austria). For mapping, we used ArcGIS ver. 9.2 (Environmental Systems Research Institute, Redlands, CA, USA), QuantumGIS ver. 2.6 (The Open Source Geospatial Foundation, Beaverton, OR, USA) and Surfer ver 12.0 (Golden Software LLC, Golden, CO, USA). The Supplementary file 2 includes the STATA code used for this study.

## 3. Results

### 3.1. Descriptive Statistics

Our analysis included data from 2478 NUTS-5 municipalities. During 1999–2012, these municipalities reported 3182 TBE cases (mean annual incidence 0.59/100,000 inhabitants). Removing non-residents from the numerator and denominator did not have a large effect on the model (see Supplementary file 3). We, therefore, left them in our analysis to improve the precision of the model estimates.

**Figure 3:**
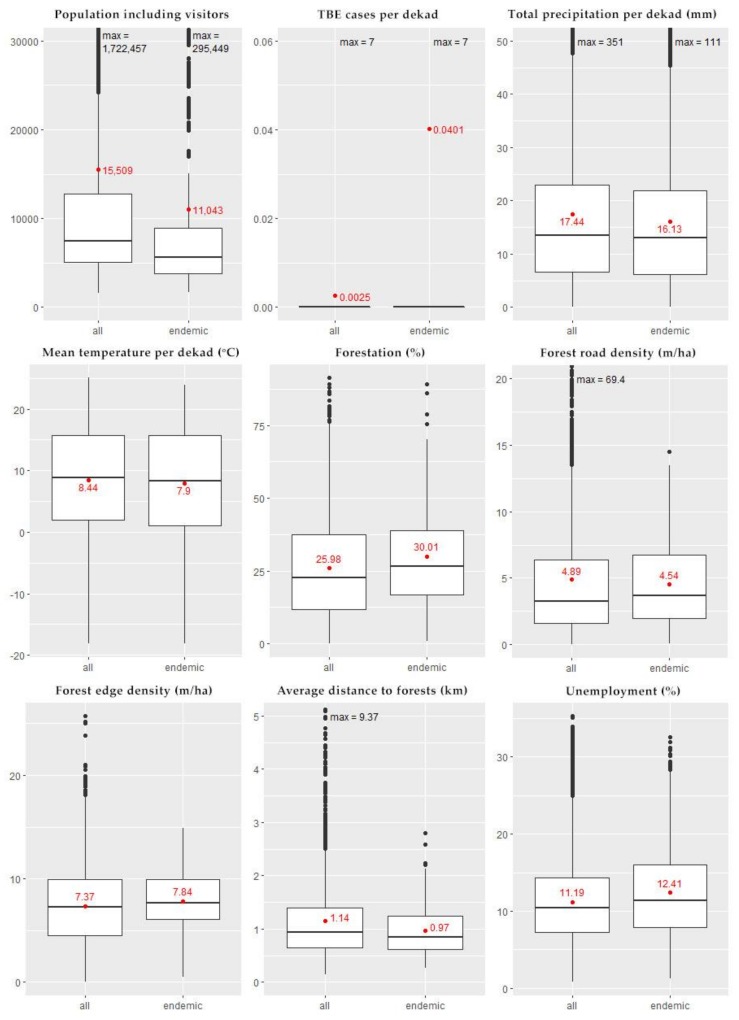
Box plots displaying the distribution of selected variables, comparing the area included in the model building (108 endemic municipalities) and the entire country territory, Poland, 1999–2012. The middle bars of the boxes show the median, and the red dots with the accompanying numbers display the mean value.

We summarized the dekad-level descriptive statistics, comparing the endemic areas in which the model was fitted with all the municipalities in Poland (Figure 3). The mean municipality population was 15,438 inhabitants (SD: 50,753; range: 1281–1,715,517), as compared to the municipality population adjusted for tourist traffic—15,509 inhabitants (SD: 50,995; range: 1595–1,722,457). Compared to the entire country, the TBE-endemic municipalities recorded lower total precipitations (16.1 mm vs. 17.4 mm), and a lower mean air temperature (7.9 °C vs. 8.4 °C).

The forested area, the length of the forest edge and length of forest roads were proportional to the NUTS-5 municipality area. Therefore, we divided these measurements by the respective municipality’s area forming, respectively, new variables: forestation, forest edge density, and forest road density. Compared to the entire country, TBE endemic municipalities had a lower forest road density (4.5 m/ha vs. 4.9 m/ha), shorter average distance to forests (0.97 km vs. 1.1 km), but higher unemployment (12.4% vs. 11.2%), larger forestation (30.0% vs. 26.0%), and higher forest edge density (7.8 m/ha vs. 7.4 m/ha).

### 3.2. Time Series Analysis

Preliminary analysis of TBE cases time series highlighted stationarity (*p*-value of Dickey-Fuller test close to 0), annual cycle (peak at frequency at periodogram about 0.028 ≈ 1/36, indicating a 36-dekad, i.e., 12-month, cycle) and strong first-order partial autocorrelation (see Supplementary file 4 for details). The observed cycle confirms the association between TBE risk and meteorological annual cycles, which is accounted for by inclusion of temperature as a predictor. Therefore, we decided to include only the first-order autocorrelation term in the final model.

Autocorrelation analyses demonstrated a very high correlation of consecutive lags in mean air temperature (around 0.9) and significant correlation of lags in precipitation (around 0.3), thus, we decided to consider only one predictor connected with each of those variables. The analysis of all possible combinations of models with various lags (see Supplementary file 5) enabled the selection of a model including precipitation with lag −3 dekads and temperature with lag −2 dekads. The decomposition of the 14-year time series indicated no longitudinal trend in TBE occurrence.

### 3.3. Variable Type Selection

All variables displayed approximate linear associations with TBE risk in log scale, aside from lagged temperature and forest edge density (see Supplementary file 6). Roughly no TBE cases were recorded in dekads preceded by negative mean air temperatures, however an increase in temperature was associated with an increase in TBE incidence. Therefore, we included an interaction term of two variables: the continuous mean temperature with lag 2 and its binary analogue indicating whether the temperature 2 dekads before was above 0. To account for the non-linear association with forest edge density, we categorized this variable into 5 intervals with equal length.

### 3.4. The Final Model

The final model (Table 2) included the same variables in the logistic and log-linear components:

1. Number of TBE cases in the previous dekad (first-order autocorrelation term);
2. Sum of precipitation three dekads before (in mm);
3. Temperature index: a binary variable indicating whether the mean air temperature two dekads before was above 0;
4. Mean air temperature two dekads before (in °C);
5. An interaction term between the above binary variable with the mean air temperature recorded two dekads before;
6. Forestation (forested area divided by municipality area, in %);
7. Forest edge density (length of forest edge divided by municipality area), categorized: 0–3 m/ha, 3–6, 6–9, 9–12, 12 and more (the most numerous category 6–9 km selected as reference level);
8. Forest road density (length of forest roads divided by the municipality area, in m/ha);
9. Average distance from settlements to forests (in kilometres); and
10. Unemployment (number of unemployed divided by population in working age, in %).

**Figure 4:**
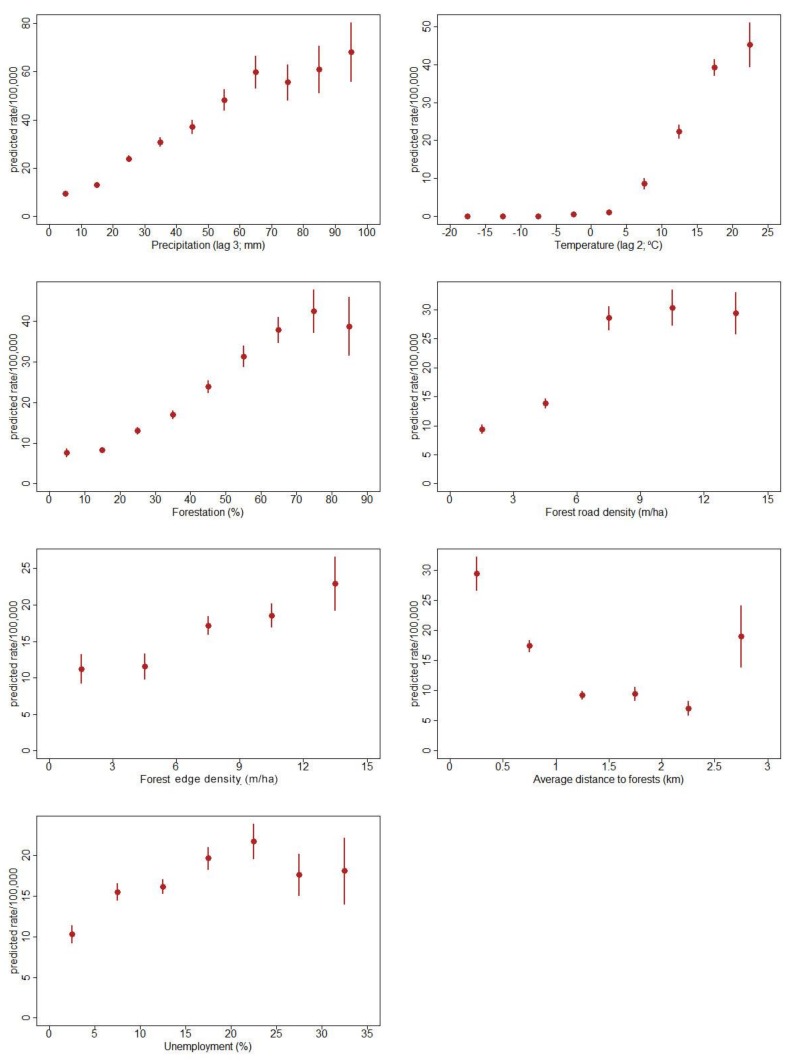
TBE predicted rates estimated at fixed values of continuous variables and stratified by categorical variable levels (forest edge density) (marginplots), 108 endemic municipalities, 1999–2012.

The coefficients in the model are included for completeness, but they do not have immediate interpretation, as the two parts of the model have to be considered in conjunction. To illustrate the impact of the covariates included in our final model, we plotted the predictions of this model at fixed values of a particular covariate and averaging over the remaining variables (Figure 4). A clear increase of incidence was predicted by higher temperature two dekads before, especially if the temperature had increased from 10 to 20 °C, and when precipitation had increased three dekads before. Incidence increased with higher forestation, when this proportion remained below 60%, flattening for the forestation exceeding 70%. Similarly, the increase of incidence associated with higher unemployment was the steepest for the unemployment below 15%, subsequently flattening out. A higher incidence was associated with higher forest road density when this indicator remained below 9 km/km^2^. Moreover, a larger forest edge and closer distance to the nearest forest also predicted higher incidence.

The $\rho^{2}$ coefficients were 0.205 for the full final model, 0.145 for the mean temperature, 0.061 for the sum of precipitation, 0.068 for the forestation, 0.051 for the forest edge density, 0.042 for the forest road density, 0.048 for the average distance to forests, and 0.041 for unemployment.

**Figure 5:**
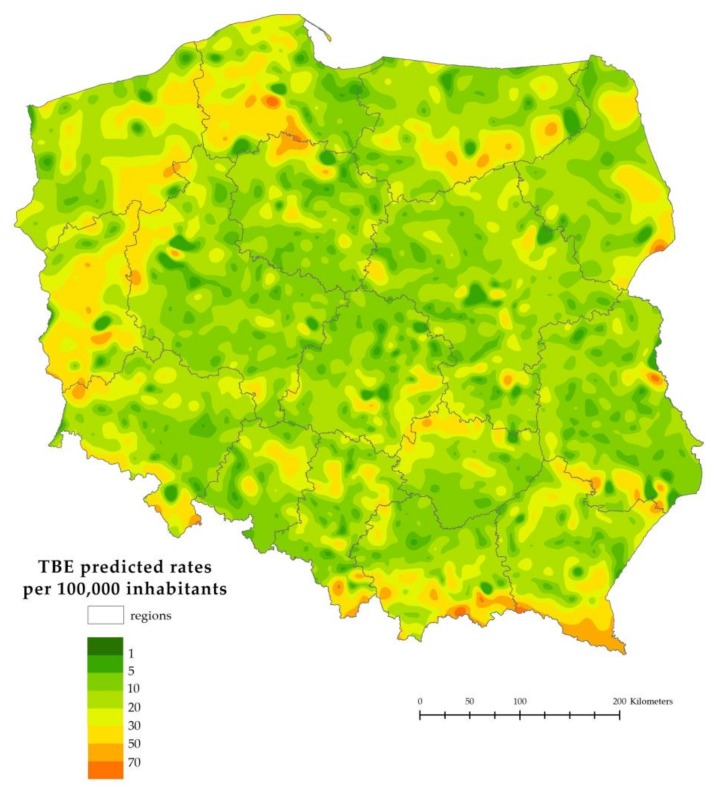
TBE rates predicted by the model per 100,000 inhabitants by NUTS-5 municipalities, Poland, 1999–2012.

The predicted TBE rates by the NUTS-5 municipality show that most areas had some predicted TBE risk, but some areas were particularly affected (Figure 5). Within the 108 endemic municipalities used for model building, the predicted rates slightly underestimated and decreased the variability as compared with the observed rates (predicted mean 16.8, SD 10.9, range 4.2–48.8 vs. observed mean 18.5, SD 16.8, range 0–81.6).

### 3.5. Validation of the Model

There was no correlation between the rates predicted at NUTS-4 level and the results of 1967–1971 surveys [15,16] (*r* = −0.0283 and −0.1382, respectively). We found a moderate correlation between the predicted rates and the 1996–2005 survey of healthy subjects [17] (*r* = 0.2816, *p* = 0.0004).

## 4. Discussion

We built a predictive model which could be applied in the future to better understand TBE risk and guide prophylactic interventions in Poland. This model, using the highest resolution available in the routinely-collected administrative data, accommodated both highly variable measurements (meteorological conditions, TBE cases occurrence), less dynamically changing socio-economic status (unemployment), and variables fairly stable in time (land use, measures of forest accessibility). In order to avoid the surveillance artefact, we trained the model using data only from the region where TBE testing was available and accessed by clinicians [20]. Still, we argue that the prediction could be extended countrywide.

We predicted high rates in most previously-established endemic areas. This was anticipated since the occurrence of TBE was an important component of the predictive model. There was, however, heterogeneity in the spatial distribution of the predicted rates even in known endemic areas. The model produced the highest predicted rates in some areas within the known endemic region, where the conditions were especially favourable for tick activity and facilitated contact between humans and ticks, rather than in areas with the highest observed TBE rates. While this could represent the lack of fit of the model, we should also acknowledge that assigning a case to a particular municipality relies on the history of travel and it may be, to some extent, arbitrary, with potential misclassification between neighbouring communes.

Factors determining TBE endemicity identified by our model are biologically plausible. High forestation, soil humidity, and air temperature increasing over the *I. ricinus* nymphal activity threshold, favour tick activity. On the other hand, higher forest accessibility (distance to forests, forest edge density, and forest roads density), increased temperatures and higher unemployment favour outdoor human activity within the tick habitats. These factors were investigated previously and confirmed as predicting TBE incidence in known endemic areas. Heterogeneous results of these studies suggest, however, that the predictive factors may be country specific, with a prominent role of land use and socio-economic determinants [4,7,9,10,12].

We predicted high TBE rates in areas considered previously as free of TBE. There is no valid explanation for the persistence of TBE foci in such limited regions as apparent from the case-based surveillance. In contrast to some countries reporting limited TBE foci, like the Scandinavian countries or Italy, where the latitudinal extent results in contrasted environmental and climatic conditions, Poland has a temperate climate and a fairly uniform geography, consisting of an almost unbroken plain reaching from the Baltic Sea in the north, to the Carpathian Mountains in the south. Most of Poland’s territory has suitable tick habitats, illustrated, for example, by the distribution of forested areas (Figure S1 in the Supplementary file 1). Many investigations carried out across the country confirmed the abundance of *I. ricinus* ticks in virtually all Polish regions, including highly urbanized ones [29,30,31,32,33]. What is making the regions that report numerous cases different is the access to TBE serological testing. In the TBE endemic areas, physicians uniformly and universally test all suspect meningo-encephalitis cases for TBE, and in the non-endemic areas, testing is seldom done. Accordingly, we believe that if suspect cases of meningo-encephalitis are not routinely referred for TBE testing in all regions in a country, the TBE reported rates cannot be considered a valid measurement of TBE risk. Indeed, circumstantial evidence from serological surveys [15,16,17,18], some environmental investigations [32], and also from an investigation comparing TBE rates in cross-border regions of Poland and Czech Republic [34], strongly suggest that such unmonitored TBE endemic areas do exist in Poland. In fact, our predicted rates correlate with some of the areas pointed previously as high-risk by seroprevalence surveys. These areas should be targeted first for improved monitoring and diagnostics.

To prepare and correctly interpret this analysis we formed a team of researchers representing several agencies in the field of public health and environmental sciences. This interdisciplinary approach is necessary to study zoonotic diseases efficiently. Indeed, the reported human cases are often simply the tip of the iceberg of processes of unknown size and intensity. Currently, little data are collected in a systematic and uniform way on variables which would better inform this kind of model, for example, human behaviours in relation to outdoor activities, data on microclimate, soil types, relative humidity, rodent density, large mammal density, etc. Having developed a framework with the best available proxy variables, we will be able to look for more precise data in the future or advocate for their systematic collection. The increasing digitization of various registers in Poland and the possibility to use large social network data to understand human behaviours may create new opportunities to predict communicable diseases in the near future. Predictive models have proven useful to estimate the burden of disease, identify the most effective prevention strategies, or to predict outbreaks and long-term trends. However, this can also be a useful exercise to better understand the data sources (i.e., surveillance systems), identify data gaps, and stimulate the extension of the research teams to include more disciplines, as well as data sources. Previous TBE predictive models were either restricted to small areas having access to large amounts of high-quality measurements [4,9,10,12] or were using remotely-sensed data [35]. In the latter case, the authors were drawing broad conclusions for Europe, which may not address local discrepancies and human behaviour peculiarities. We think that predictive models cannot replace real data in making public health decisions, but can be a valuable addition, if interpreted carefully.

Our analysis has several limitations. First, our model could suffer from “ecologic bias”, if it missed determinants of TBEV survival that affect processes at a much finer spatial resolution. For example, previous investigations have shown that air temperatures measured at 2 m above ground do not necessarily reflect the microclimate of tick habitats, and the sum of precipitation displays poor correlation with atmospheric saturation deficit and relative humidity, which are key determinants of tick activity [13]. In addition, we did not consider any direct measurements of human activity or animal populations. This can explain the moderate value of McFadden’s *R*^2^ (0.205) for the full model. Our analysis was not aimed, however, at explaining the local biological processes, but rather an attempt to use routinely-collected measurements as proxy indicators influencing the local microclimate, and to identify a broad-scale set of determinants that makes high-risk TBE areas different from areas with non-existing or lower risk. The fit of the model and strong effects of meteorological variables indicate that this approach is justified. Second, the assignment of TBE cases to the municipality of exposure may be biased by its non-standardized recording in the surveillance forms. If the interviewer misses information on recent travel, the case could be wrongly assigned to its residence address. Additionally, travel to multiple locations during the exposure period, or travel abroad, could lead to misclassification. Since most cases are among residents of rural areas, they are likely mostly exposed in their residence municipality, compared to urban dwellers. Furthermore, only 11.4% of TBE cases had recorded a place of exposure outside of their municipality of residence. We, therefore, believe the effect of such misclassification to be limited in our analysis. Third, our population denominator was likely underestimated, because the number of bed-days of tourist accommodations does not reflect all visitors (i.e., those visiting for one day, tourists sleeping in tents or in unregistered accommodations). Our adjustment of population denominator, although largely underestimated, permitted differentiation between highly popular tourist destinations from the rest. The underestimated adjustments for tourists should not have a major impact on our analysis, which was supported by the results of our sensitivity analysis (Supplementary file 3). Fourth, the land use measures were only calculated once, with the assumption that they do not change over time. This is not completely accurate, as there may be changes in the forest structure which may affect tick habitats and forest accessibility. If we would choose to study longer time series of data, we would definitely have to account for annual changes in the land use, possibly involving agencies collecting such data. For a 14-year period we can however assume a relative stability of land use. Fifth, an important limitation of our analysis is the lack of proper model validation. The historical seroprevalence data were assigned to larger areas than NUTS-4 district because of insufficient sample sizes. Since we are missing the case-based results of the old surveys, we had to assign values to particular districts from low-resolution maps. Additionally, the oldest surveys were using the complement fixation test, which had higher specificity but low sensitivity, compared to the IgG ELISA test. This could lead to underestimation of these surveys results, as compared to the newer ones.

## 5. Conclusions

Our study permitted identification of regions in Poland where a set of modifiable and non-modifiable factors favoured persistence of TBE virus and increased TBE risk. On the basis of our results, supported by available circumstantial evidence, we can conclude that TBE is currently not appropriately monitored. Our predicted TBE risk map is partly consistent with high risk areas identified by surveillance, but high TBE risk was also predicted in many areas where no TBE cases were reported over the past three dekads.

We recommend raising awareness amongst doctors in the areas with the highest predicted incidence. Additionally, our results should be validated by ensuring the availability of TBE diagnosis in all Polish hospitals, and considering adding alternative sources of data on TBE risk, for example, routine household animal or wildlife surveys. We also recommend increasing the capacity in infectious disease modelling in the field of Public Health field by bringing more expertise, and new data sources, for routine monitoring of zoonosis risk, understanding data gaps, and identifying the most efficient prevention measures.

## Figures and Tables

**Table 1:** Characterisation of variables preparation for the predictive model, Poland, 1999–2012.

Variable Description	Granularity	Unit	Source	Data Processing
Number of TBE cases	By dekaddekad of onset	Count	National Institute of Public Health	We assigned notified cases to their municipality of exposure, by dekaddekad of onset.
Population denominator	By year	Count	Central Statistical Office	For each municipality, we obtained the population estimates on the 30 June of each year. Since we assigned cases by municipality of exposure, the numerator included both residents and tourists. Therefore, we added to the denominator the estimated number of visiting tourists (Polish nationals), based on the Central Statistical Office estimate of the number of bed-days occupied by visitors, divided the number of days in a year. For 1999–2003, we imputed the proportions of municipality population increases.
Mean temperature	By dekaddekad	°C	Institute of Meteorology	We used mean daily air temperature measurements from 54 synoptic weather stations evenly distributed in Poland. To assign measurements to each municipality, we used residual kriging—a spatial interpolation method [21,22] previously validated for use with Polish meteorological data [23]. For each dekaddekad, we calculated the mean temperature and created a raster map at 250 m spatial resolution, including the values interpolated from the 54 stations. Then we used a vector map of NUTS-5 boundaries to assign the average value to each municipality.
Sum of precipitation	By dekaddekad	Mm	Institute of Meteorology	We used daily sum of precipitation measurements from 54 meteorological stations. To assign measurements to each municipality, we used co-kriging, recommended when spatial correlation is found between covariables and the variable of interest and when the covariables are oversampled with respect to the primary variable [21]. The method was previously validated with Polish data [24]. For each dekaddekad, we calculated the total precipitation and created a raster map at 250 m spatial resolution, including the values interpolated from the 54 stations. Then we used a vector map of NUTS-5 boundaries to assign the average value to each municipality.
Unemployed	By year	Count	Central Statistical Office	Data at the municipality level on the number of registered unemployed were available for 2003–2012. For 1999–2002, we imputed these numbers to each municipality based on the numbers recorded in districts (NUTS-4), according to the proportional distribution between municipalities forming each district, as observed during 2003–2012.
Forested area	Calculated once for study period	Ha	CORINE Land Cover 2006 NUTS-5 boundaries	We merged all polygons representing forest classes (CLC code 3.1). We intersected the forest layer with the map of NUTS-5 administrative boundaries to obtain the area of forests contained in each municipality.
Length of forest edge	Calculated once for study period	Km	CORINE Land Cover 2006 NUTS-5 boundaries	Using the above described forest layer, we converted the forest polygons to lines. Then we intersected the resulting layer with the NUTS-5 administrative boundaries. We excluded segments overlaying with the municipality boundaries or located within a 50 m buffer, to account for the results of the intersection between forest edges with administrative boundaries. Then we computed the remaining length of lines for each municipality in km.
Average distance from settlements to forests	Calculated once for study period	Km	CORINE Land Cover 2006 IMAGIS settlement map NUTS-5 boundaries	We used the proximity (raster distance) function of QGIS to calculate the distances between forests (from CORINE CLC 3.1) at 100 m resolution. Then we converted the data raster into a polygon distance layer, where each 100 × 100 m polygon had an attribute describing the distance from the nearest forest. Next, we intersected the above described distance polygon layer with two complementary maps: the polygon CORINE map (CLC code 1.1 urban fabric), containing more precise information on urban settlements and a more detailed point map of smaller settlements (after deleting points overlapping with urban fabric polygons). We intersected both maps with the polygon distance layer, and calculated the average distance from settlements to forests, using the mean of both values for each municipality.
Length of forest roads	Calculated once for study period	Km	CORINE Land Cover 2006 www.geofabrik.de/ NUTS-5 boundaries	We intersected the layer containing the road network with the CORINE map of forests (CLC 3.1) and with the NUTS-5 boundaries. We extracted all types of roads crossing the forests polygons. We calculated the total length in km in each municipality.

NOTE: A “dekad” is a 10-day period.

**Table 2:** Final model assessing the associations between determinants of TBE endemicity with the TBE occurrence, 108 endemic municipalities, 1999–2012.

Variable	Coefficient	Level of Significance
LOG-LINEAR PART		
Number of TBE cases (−1 dekad)	**0.215**	***
Sum of precipitation (−3 dekads)	**0.009**	***
Temperature index (if > 0 °C)	**2.071**	***
Mean temperature (−2 dekads)	−0.227	NS
Interaction (temp. index × mean temp.)	0.203	NS
Forestation	**0.036**	***
Forest edge density (ref: 6–9 m/ha)	-	-
0–3	0.321	NS
3–6	**−0.505**	***
9–12	**0.299**	**
>12	**0.736**	***
Forest road density	**−0.059**	**
Average distance to forests	0.139	NS
Unemployment	**0.047**	***
Constant in the model	**−11.197**	***
LOGISTIC PART		
Number of TBE cases (−1 dekad)	**−0.839**	***
Sum of precipitation (−3 dekads)	−0.010	*
Temperature index (if >0 °C)	1.600	*
Mean temperature (−2 dekads)	**−0.493**	***
Interaction (temp. index * mean temp.)	0.219	NS
Forestation	**0.032**	***
Forest edge density (ref: 6–9 m/ha)	-	-
0–3	0.148	NS
3–6	−0.374	NS
9–12	0.472	*
>12	**1.074**	***
Forest road density	**−0.194**	***
Average distance to forests	0.568	NS
Unemployment	**0.072**	***
Constant in the model	0.704	NS

Levels of significance: NS—*p* > 0.05; * 0.05 > *p* > 0.01; ** 0.01 > *p* > 0.001; *** *p* < 0.001. In bold are variables significant at level *p* < 0.01.

## Supplementary Materials

The following are available online at http://www.mdpi.com/1660-4601/15/4/677/s1; Supplementary file 1: preparation of variables, Supplementary file 2: selected STATA code, Supplementary file 3: comparison of the final model with all cases with a model excluding imported cases, Supplementary file 4: results of the preliminary analysis of time series with number of cases reported from endemic municipalities, Supplementary file 5: selection of lags for the meteorological measurements, Supplementary file 6: checking for the possibility to use independent variables as continuous in the log-linear model, Figure S1: map of CORINE landcover forest classes (CLC code 3.1) areas, Poland, 2006.
