# Supplementary material for: A Predictive Model Has Identified Tick-Borne Encephalitis High-Risk Areas in Regions Where No Cases Were Reported Previously, Poland, 1999–2012
Source: Int J Environ Res Public Health. 2018 Apr 4;15(4):677. doi: 10.3390/ijerph15040677 (PMC5923719; doi:10.3390/ijerph15040677)
Supplement: Supplementary file 1 [file ijerph-15-00677-s001.zip › ijerph-281680-supplementary/Supplementary file 1.docx]

**Supplementary materials for manuscript “A predictive model identified tick borne encephalitis high risk areas in regions were no cases were reported previously, Poland, 1999-2012”**

**Text S1. Preparation of variables**

Below we describe in detail how each variable was prepared.

*1.1. GIS variables*

*1.1.1. Mean air temperatures interpolated to country area*

Source data:

- Mean daily air temperatures originated from 54 synoptic weather stations (from the Institute of Meteorology and Water Management)
- DEM (Digital Elevation Model) map layer (from U.S. Geological Survey (2011), <http://dds.cr.usgs.gov/srtm/>)
- Map of NUTS-5 administrative boundaries (from the National Register of Boundaries, <http://www.geoportal.gov.pl/>)

Software used:

- ArcGIS package ver. 9.2
- Surfer ver. 12.0

We obtained mean daily air temperatures recorded in 54 synoptic weather stations, located uniformly in the country. These stations measure in standard way air temperature at two metres above ground level, at synoptic times, every three hours (totally eight terms per day) and calculate the mean daily temperature, using a simple arithmetic mean of the eight measurements. The mean dekadal temperature is calculated by summing mean daily measurements and dividing by 10.

We mapped the mean dekadal temperatures using coordinates of synoptic stations, and interpolating the intermediate values for Poland territory, using residual kriging. This approach was previously validated for interpolation of meteorological data in Poland. In the first step, multivariate linear regression (formula 1) is fitted between the spatial variable *Z*(s) - the spatially-referenced temperature measurements - and the set of explanatory variables *x_i_*(s), including the elevation, inclination, aspect, distance to coast, latitude (from DEM map). The residuals of regression model *δ*(s) are calculated for each sample location and interpolated using ordinary kriging method.

| 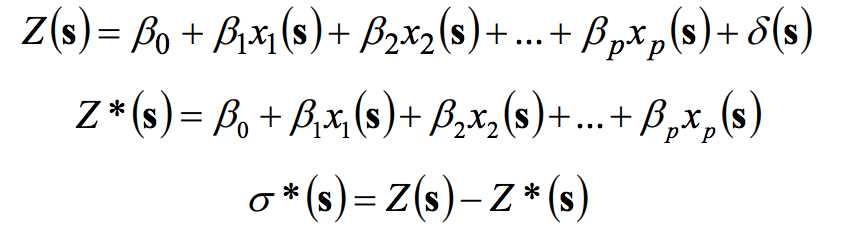 | (1) |
| --- | --- |

In the second step residual values *δ*(s) are assumed as a random variable that follows the intrinsic hypothesis and values of *δ**(s) are interpolated by ordinary kriging. The final predicted values of Z(s) are obtained by summation of *Z**(s) and *δ**(s) over the kriging grid.

The full statistical procedure is described in [1,2]. It was previously validated with Polish data and deemed appropriate for routine interpolation of mean temperature measurements [3].

After obtaining a raster map of interpolated values of mean dekadal measurements in 250 metres resolution, we extracted the average values from the raster map using the NUTS-5 administrative boundaries (status from 2005). We repeated this procedure for each dekad, thus obtaining 504 map layers (36 dekads x 14 years).

*1.1.2. Sum of precipitation interpolated to country area*

Source data:

- Sum of daily precipitation recorded in 54 synoptic weather stations (from the Institute of Meteorology and Water Management)
- DEM (Digital Elevation Model) map layer (from U.S. Geological Survey (2011), <http://dds.cr.usgs.gov/srtm/>
- Map of NUTS-5 administrative boundaries (from the National Register of Boundaries, <http://www.geoportal.gov.pl/>)

Software used:

- ArcGIS package ver. 9.2
- Surfer ver 12.0

We obtained daily precipitation totals recorded in standard way at 54 synoptic weather stations, located uniformly in the country. The daily values were added to obtain the dekadal sum of precipitation.

We used the cokriging procedure to interpolate the data from 54 synoptic stations to the entire country area, using the DEM data as covariable. The cokriging procedure is an extension of kriging when multivariate variogram or covariance model and multivariable data are available. The precipitation measurements were cokriged at synoptic stations locations from data about itself and about DEM variables in the neighbourhood. In this approach we have to estimate the variogram model for the variable of interest, the variogram models for all covariables and also the cross-variogram models for all pairs of the primary variable and each covariable.

We chose cokriging as we expected spatial correlation between elevation data and precipitation measurements and oversampling of elevation data with respect to the precipitation measured only at 54 synoptic stations locations. This method was described in more detail [1,2] and validated with Polish data [4].

After obtaining a raster map of interpolated values of mean dekadal measurements in 250 metres resolution, we extracted the average values from the raster map using the NUTS-5 administrative boundaries (status from 2005). We repeated this procedure for each dekad, thus obtaining 504 map layers turned into datasets (36 dekads x 14 years).

*1.1.3. Forested area*

Source data:

- CORINE LandCover 2006 (scale 1:100 000, from the European Environmental Agency, <https://www.eea.europa.eu/publications/COR0-landcover>)
- Map of NUTS-5 administrative boundaries (from the National Register of Boundaries, <http://www.geoportal.gov.pl/>)

Software used:

- ArcGIS package ver. 9.2

In the CORINE LandCover map we merged all polygons representing forest classes (CLC code 3.1). We intersected these forest polygons with the map of NUTS-5 administrative boundaries, thus obtaining the area covered by forests in each municipality.

The map below (Figure S1) displays the forested areas distribution across Poland.


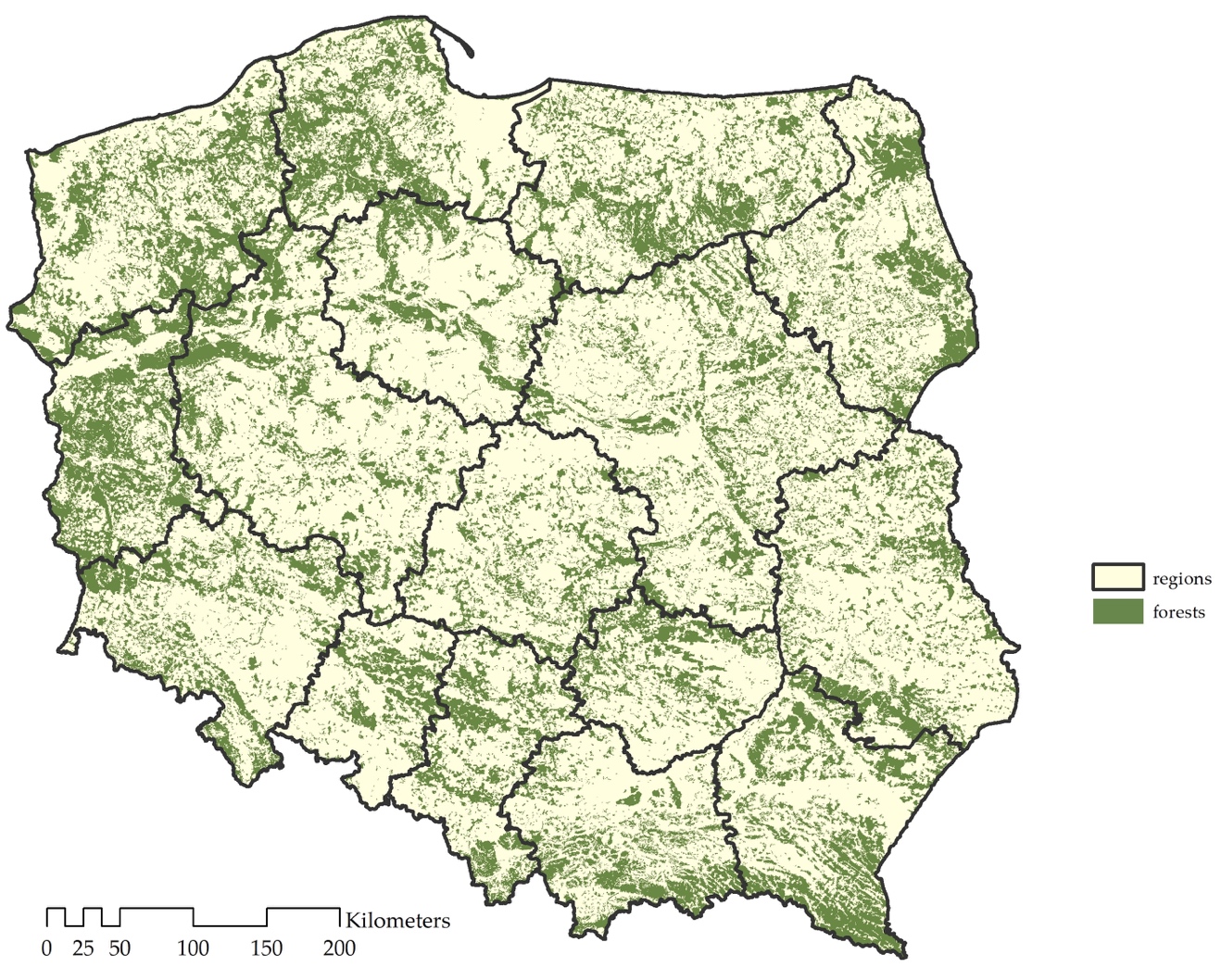


Figure S1. Map of CORINE LandCover forest classes (CLC code 3.1) areas, Poland, 2006.

*1.1.4 Length of forest edge*

Source data:

- CORINE LandCover 2006 (scale 1:100 000, from the European Environmental Agency, <https://www.eea.europa.eu/publications/COR0-landcover>)
- Map of NUTS-5 administrative boundaries (from the National Register of Boundaries, <http://www.geoportal.gov.pl/>)

Software used:

- ArcGIS package ver. 9.2

We calculated the length of the forest edge as a measure of forest fragmentation (proxy for accessibility to local inhabitants). For this calculation we intersected the CORINE LandCover map of all forest classes (CLC code 3.1) with the map of NUTS-5 administrative boundaries, to obtain a map of forest polygons contained inside respective municipalities. Then we converted the polygons to lines. Subsequently, we deleted the lines corresponding with the administrative edge (as the result of the intersection). Additionally, we wanted to consider only forest edge which were accessible from the inside of the municipality area. To account for this, we used 50-meter buffers inside the NUTS-5 polygons to delete lines that were placed in close proximity, but not exactly overlaying the municipality edge. Then we calculated the forests edge length in km using the standard function in ArcGIS.

*1.1.5. Average distance from settlements to forests*

Source data:

- CORINE LandCover 2006 (scale 1:100 000, from the European Environmental Agency, <https://www.eea.europa.eu/publications/COR0-landcover>)
- Map of 50,000 settlements (from IMAGIS, <http://imagis.pl/en/>)
- Map of NUTS-5 administrative boundaries (from the National Register of Boundaries, <http://www.geoportal.gov.pl/>)

Software used:

- ArcGIS package ver. 9.2
- Quantum GIS ver. 2.6
- MS Access

Living in proximity to forests has been identified as a major risk factor in many previous investigations. To calculate the average distance from settlements to the forests, we first mapped the distances between forest polygons in the CORINE LandCover map of all forest classes (CLC 3.1). We used the proximity (raster distance) function of Quantum GIS software to create a raster layer representing in defined resolution distance from particular objects.

The raster distance was generated separately for each of the sixteen Polish regions, to account for convenient workflow and less data processing consumption (Figure S2). We set the raster data resolution at 100 meters. The higher resolution would result in longer data processing and in some cases end up in PC errors, therefore 100 meters was a satisfactory compromise between data precision and data time processing.


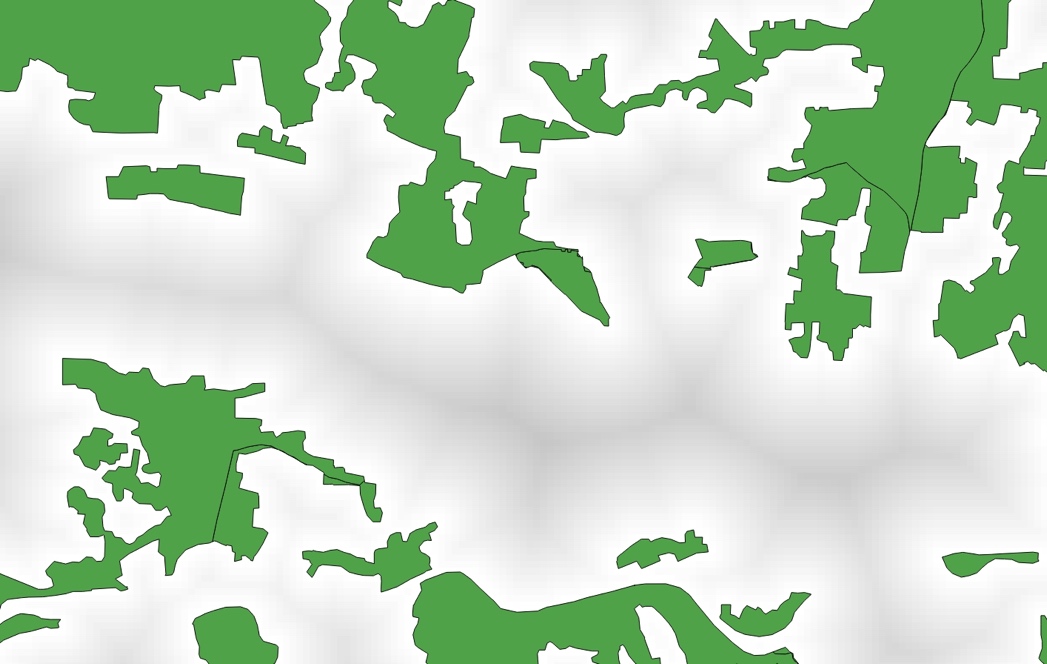


Figure S2. Example of the QGIS proximity raster result. The green polygons are forests and the shades of grey reflect the distance from the forests.

Then we converted the raster data into a polygon layer. In this map each 100x100 polygon had an attribute describing the distance from the nearest forest edge. The software had automatically merged the neighbouring polygons with the same distance attribute values, making their shape irregular (not 100x100 squares like in the raster file, Figure S3).


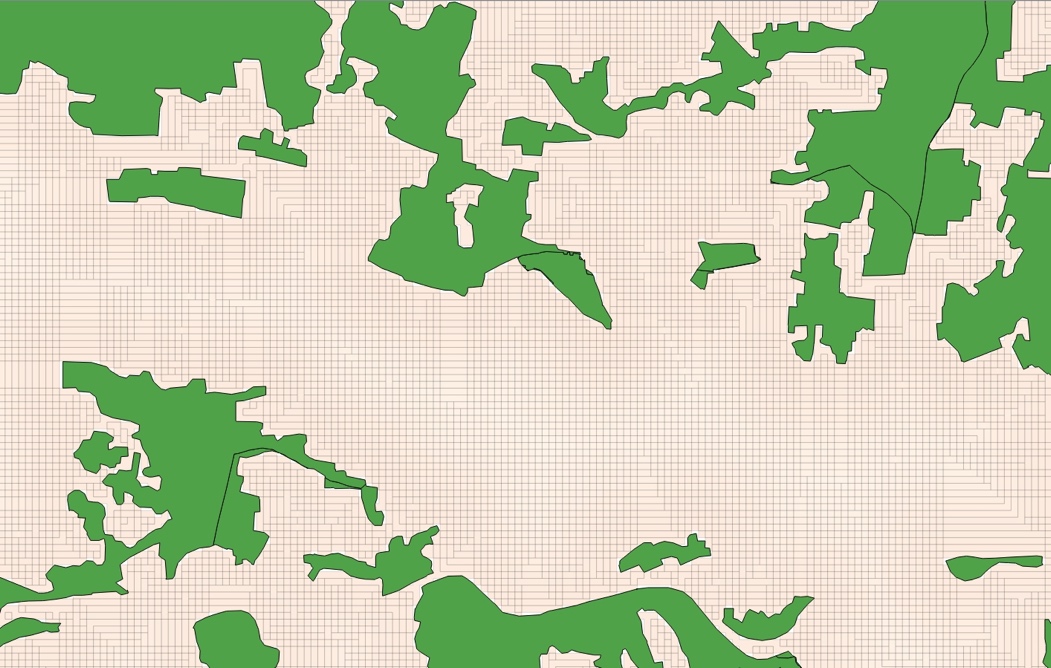


Figure S3. QGIS proximity raster to polygon result: Green polygons are forests; Beige polygons contain distance information transformed from the raster file (rectangles of different sizes are merged 100x100 metres squares with the same value of the distance attribute).

Next, we intersected the CORINE LandCover map (CLC code 1.1 Urban fabric) with the above distance polygon layer to calculate an average distance from residential areas to the nearest forest. We intersected the resulting layer with the maps of NUTS-5 administrative boundaries to calculate the average distance by municipality (Figure S4). Since the polygons in the distance polygon layer did not have regular shapes, we decided to calculate the mean distances weighted by the urban fabric area.


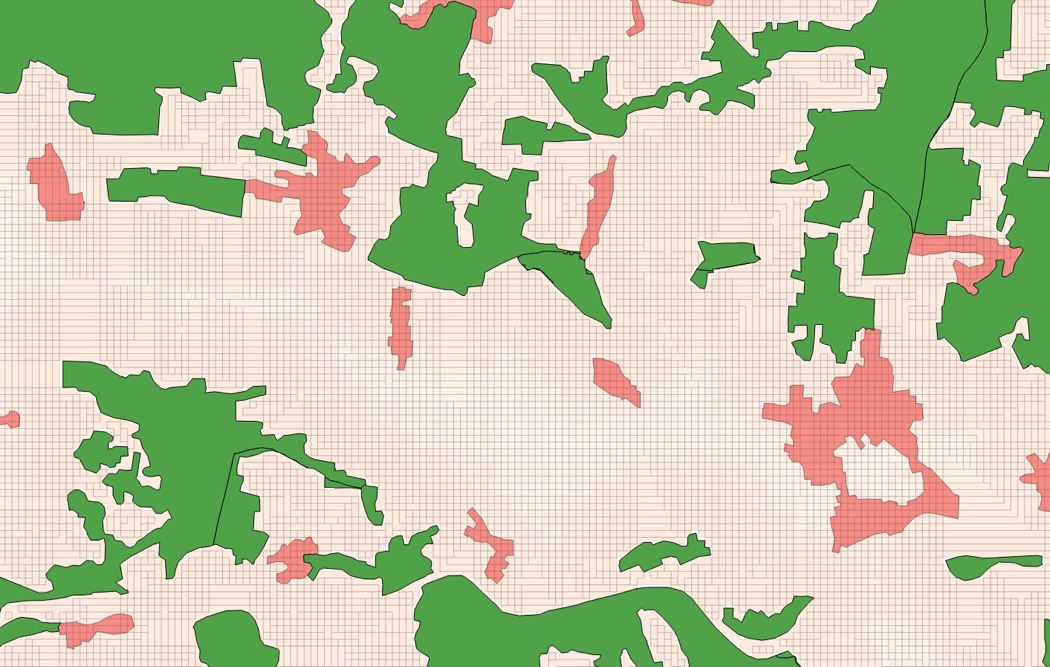


Figure S4. Polygon map with the forests and residential areas (urban fabric): Green polygons are forests; Pink polygons are residential areas; Beige rectangles are distance polygons (as described above).

The CORINE LandCover urban fabric map was incomplete with regards to smaller settlements. We therefore used an additional point map of settlements to obtain more comprehensive location of residential locations. We decided to use both maps because they were complimentary. The urban fabric polygon map reflected more precisely the area and extent of larger settlements and towns, and the point map of settlements reflected the location of smaller settlements like isolated farms. Using only the more complete point map would introduce large errors in distance calculations, as it depicts even large towns as points.

After overlaying both maps, we removed all points intersecting with CLC urban fabric areas. Then we repeated the distance calculation for each point, by intersecting the point map of settlements with the distance polygon distance layer, and subsequently with the NUTS-5 boundaries layer.

Then we added the values obtained from both calculations in an MS Access dataset and recalculated the average distances from settlements to forests for each municipality.

*1.1.6. Length of forest roads*

Source data:

- CORINE LandCover 2006 (scale 1:100 000, from the European Environmental Agency, <https://www.eea.europa.eu/publications/COR0-landcover>)
- Road network map (from [www.geofabrik.de/](http://www.geofabrik.de/))
- Map of NUTS-5 administrative boundaries (from the National Register of Boundaries, <http://www.geoportal.gov.pl/>)

Software used:

- ArcGIS package ver. 9.2

The density of forest roads is an important indicator of accessibility of tick habitats to local inhabitants visiting forested areas. First, we intersected the road network map with the CORINE LandCover map of forests (CLC classes 3.1) to extract all types of roads that are contained within the forests polygons. Then, we intersected the resulting layer of forest roads with the map of NUTS-5 administrative boundaries, to calculate the total length of roads in km by municipality.

*1.2. National statistics*

*1.2.1. Number of inhabitants by municipality (population denominator for model)*

Source of data: Local Data Bank, Central Statistical Office (<https://bdl.stat.gov.pl/BDL/start>)

We used the population estimates published by the National Statistical Office each year on the 30^th^ June, available for each NUTS-5 municipality. Because during the studied period there were changes in the NUTS-5 administrative division, we adopted one reference set of NUTS units, based on the status in 2005 (roughly in the middle of the studied period). Subsequently, if an administrative unit was cancelled, renamed or merged with another one, we assigned the values to the corresponding reference units. All changes in the administrative division are listed on this page: <https://bdl.stat.gov.pl/BDL/metadane/teryt/zmiany>)

To account for the changing population during the tourist season (increase of at risk population for tick exposure), we added the estimated number of tourists to the resident population estimates. For this, we used the number of bed-days occupied, as recorded for tax purposes. We divided the number of bed-days in particular municipality in given year by number of days in that year (365 or 366) and added it to local population count to receive approximate overall population count adjusted by visitors activity (details below in section S1.3.1.).

*1.2.2. Number of TBE cases by municipality and dekad*

Source of data: TBE register, National Institute of Public Health – National Institute of Hygiene

We used the information from case reports filled for every reported TBE case, meeting the surveillance case definition. We assigned cases to NUTS-5 municipalities by place of exposure. For each case, we extracted the information on probable place of exposure and assigned to a NUTS-5 municipality. If there was no information on travel, we assumed that the case was exposed locally, in their municipality of residence. We used the consistently recorded date of disease onset to assign cases to respective dekad (10-day period).

*1.2.3. Unemployment*

Source of data: Local Data Bank, Central Statistical Office (<https://bdl.stat.gov.pl/BDL/start>)

We used the information on the number of registered unemployed, recorded by the Central Statistical Office. For 1999-2002, data were not available at NUTS-5 level (only at NUTS-4 level). Therefore, we imputed the missing records, as describe in detail below, in the section S1.3.2.

We calculated the unemployment ratio by dividing the number of unemployed by the estimated population professionally active (18-64 years for men and 18-59 years for women).

*1.3. Data imputation*

For two variables obtained from the Central Statistical Office, data were not available at NUTS-5 level for the entire study period. Below we describe in detail how we imputed the missing values.

We defined:

$k$ – municipality index

$d(k)$ – district containing municipality $k$

$i$ – year index

*1.3.1. Population adjusted by number of visitors*

Data on bed-days used by visitors were available for years $i=2004,\ldots,2012$. Therefore, we calculated visitor-adjusted population for these years as a sum of local population count and overall bed-days used by visitors divided by number of days in a certain year, using formula (2):

| $P_{v}^{i,k}=P_{l}^{i,k}+\frac{b^{i,k}}{d^{i}}$ $i=2004,\ldots,2012$ | (2) |
| --- | --- |

Where:

$P_{v}^{i,k}$ – population adjusted by number of visitors (year $i$, municipality $k$)

$P_{l}^{i,k}$ – local population (year $i$, municipality $k$)

$b^{i,k}$ – overall bed-days used by visitors (year $i$, municipality $k$)

$d^{i}$ – number of days in year $i$, equal 365 or 366

Data on bed-days used by visitors were NOT available for years $i=1999,\ldots,2003$. Therefore, we used formula 3 to estimate the average population increase, based on the trends in consecutive years. We multiplied the local population count by average population increase (after adjusting for visitors) received for years 2004-2012, for which data were available.

| $P_{v}^{i,k}=P_{l}^{i,k}\cdot r_{v}^{k}=P_{l}^{i,k}\cdot\frac{\sum_{j=2004}^{2012} P_{v}^{j,k}}{\sum_{j=2004}^{2012} P_{l}^{j,k}}$ $i=1999,\ldots,2003$ | (3) |
| --- | --- |

Where $r_{v}^{k}$ – rate of average increase in population (in years $2004,\ldots,2012$) after adjusting by number of visitors (municipality $k$).

*1.3.2. Unemployment ratio*

To calculate the unemployment ratio, we divided number of unemployed people by count of population in productive age, using the formula 4:

| $r_{u}^{i,k}=\frac{P_{u}^{i,k}}{P_{p}^{i,k}}\cdot100\%$ , $i=1999,\ldots,2012$ | (4) |
| --- | --- |

Where:

$r_{u}^{i,k}$ – unemployment ratio in % (year $i$, municipality $k$)

$P_{u}^{i,k}$ – population unemployed (year $i$, municipality $k$)

$P_{p}^{i,k}$ – population in productive age (year $i$, municipality $k$)

For years $i=2003,\ldots,2012$ data on unemployed population was available at NUTS-5 level. Since the $P_{u}^{i,k}$ was known, we just included it into formula 4.

For years $i=1999,\ldots,2002$ data on unemployed population was available at NUTS-4 level only. Therefore, we have estimated the number of unemployed using the proportional distribution of unemployed between municipalities included in respective districts during consecutive years (formula 5).

| $P_{u}^{i,k}=P_{u}^{i,d(k)}\cdot p_{d\left( k \right)}^{k}=P_{u}^{i,d\left( k \right)}\cdot\frac{\sum_{j=2003}^{2012} P_{u}^{j,k}}{\sum_{n\in d\left( k \right)} \sum_{j=2003}^{2012} P_{u}^{j,n}}$, $i=1999,\ldots,2012$ | (5) |
| --- | --- |

Where:

$P_{u}^{i,d(k)}$ – number of unemployed in a district containing municipality $k$ (year $i$)

$p_{d(k)}^{k}$ – mean proportion of unemployed from district containing municipality $k$ assigned to municipality $k$

$\sum_{n\in d\left( k \right)} \sum_{j=2003}^{2012} P_{u}^{j,n}$ – sum of number of unemployed in years $j=2003,\ldots,2012$ in municipalities included in district containing municipality $k$

**References**

1. Hofstra, N.; Haylock, M.; New, M.; Jones, P.; Frei, C. Comparison of six methods for the interpolation of daily, European climate data. *Journal of Geophysical Research: Atmospheres* **2008**, *113*, D05109.
2. Tveito, O. E.; Wegehenkel, M.; van der Wel, F.; Dobesch, H. *The Use of Geographic Information Systems in Climatology and Meteorology*; Office for Official Publications of the European Communities, 2008.
3. Ustrnul, Z.; Czekierda, D. Application of GIS for the development of climatological air temperature maps: an example from Poland. *Meteorological Applications* **2005**, *12*, 43–50.
4. Lupikasza, E.; Ustrnul, Z.; Czekierda, D. The role of explanatory variables in spatial interpolation of selected climate elements. *Annals of Geomatics* **2007**, *7*, 55–68.
