# Supplementary material for: A Predictive Model Has Identified Tick-Borne Encephalitis High-Risk Areas in Regions Where No Cases Were Reported Previously, Poland, 1999–2012
Source: Int J Environ Res Public Health. 2018 Apr 4;15(4):677. doi: 10.3390/ijerph15040677 (PMC5923719; doi:10.3390/ijerph15040677)
Supplement: Supplementary file 1 [file ijerph-15-00677-s001.zip › ijerph-281680-supplementary/Supplementary file 2.docx]

**Supplementary materials for manuscript “A predictive model identified tick borne encephalitis high risk areas in regions were no cases were reported previously, Poland, 1999-2012”**

**Text S2. Selected STATA code**

/* We’ve been using separate csv file for each variable. */

/* Files with data for model building (17 districts) had 504 rows (14 years times 36 dekads gives 504 dekads) and 108 columns (number of municipalities in selected 17 districts). */

/* Files with data for predicting (all districts) had 504 rows and 2478 columns (number of all municipalities in Poland). */

// CONSTRUCTING THE DATASETS

// Number of cases

insheet using "~\TBE\cases17districts.csv", delimiter(";") names clear

reshape long v, i(dekad) j(id_municipality)

sort id_municipality dekad

save TBE_model_data

// Temperature

insheet using ""~\TBE\temp17districts.csv", delimiter(";") names clear

reshape long v, i(dekad) j(id_municipality)

sort id_municipality dekad

merge 1:1 id_municipality dekad using TBE_data, generate(m)

drop m

save TBE_model_data, replace

// et cetera...

/* Setting the panel time series structure to complete dataset (with all variables) */

egen id_dekad = group(dekad)

xtset id_municipality id_dekad

/* Both datasets (TBE_model_data with 108 municipalities and TBE_pred_data with all municipalities) were constructed in a way presented above. */

/* List of variables before the transformations:

cases – number of TBE cases

temp – mean temperature

rain – sum of precipitation

forest_area – forest area

forest_border – length of forest borders

forest_road – length of forest roads

forest_dist – mean distance to forests

unemp – unemployment

Each of above variables is a panel time series variable. */

/* DATA TRANSFORMATIONS, PRELIMINARY ANALYSIS, LAGS SELECTION AND LINEARITY CHECK */

// Autocorrelation terms and meteorological variables with lags

gen L1cases = L.cases

gen L1temp = L.temp

gen L1rain = L.rain

gen L2cases = L2.cases

gen L2temp = L2.temp

gen L2rain = L2.rain

// et cetera...

// Logarithm of population for ZIP model

gen logpop = log(pop)

/* Preliminary analysis of time series with sums of cases from endemic municipalities */

insheet using ""~\TBE\sum_cases17.csv", delimiter(";") names clear

egen id_dekad = group(dekad)

tsset id_dekad

pergram endemic_cases

pac endemic_cases

dfuller endemic_cases , lags (1) drift regress

// Lags selection

/* Procedure: build a simple zip model for each combination of lags (equal 1, 2, 3 or 4) for autocorrelation term, precipitation and temperature, check AIC and BIC for each model, select combination of lags with lowest AIC and BIC. */

/* Exemplary model with lags equal 2 and 3 for precipitation and temperature, respectively and 1-order autocorrelation term */

zip cases (L1cases L2rain L3temp), inflate(L1cases L2rain L3temp) offset(logpop)

estat ic

// Linearity check

/* Do the following procedure for each predictor v:

1) categorize v: cut into intervals of equal length,

2) build a full zip model m with all other predictors continuous and v categorical,

3) plot logarithm of predictions from model m for consecutive levels of v (other variables set to their means) and check whether the trend is linear. */

/* Example of linearity check for precipitation */

egen L3rain_cat = cut(L3rain), at(0,20,40,60,80,100,120)

zip cases (L1cases i.L3rain_cat L2temp forest_area forest_border forest_road forest_dist unemp), inflate(L1cases i.L3rain_cat L2temp forest_area forest_border forest_road forest_dist unemp) offset(logpop)

estimates save Model_lin.ster

use "~\TBE\pred_data.dta", clear \\ dataset with means for prediction

replace L3rain = 10 in 1

replace L3rain = 30 in 2

replace L3rain = 50 in 3

replace L3rain = 70 in 4

replace L3rain = 90 in 5

replace L3rain = 110 in 6

egen L3rain_cat = cut(L3rain), at(0,20,40,60,80,100,120)

estimates use Model_lin

predict pred // first 6 rows of pred

gen logpred = log(pred)

// and then check if first 6 values of logpred show linear trend

// Categorical variables

egen forest_border_cat = cut(forest_border), at(0, 3, 6, 9, 12, 26)

egen L2temp_cat = cut(L2temp), at(-20, 0,26) icodes

save TBE_model_data, replace

/* Then the variables L1cases, L3rain, L2temp, L2temp_cat and forest_border_cat were constructed in TBE_pred_data dataset. */

// FINAL MODEL BUILDING, PREDICTION AND MARGINS

// Final model

use "~\TBE\TBE_model_data.dta", clear

zip cases (L1cases L3rain i.L2temp_cat L2temp_cat##c.L2temp forest_area ib6.forest_border_cat forest_road forest_dist unemp), inflate(L1cases L3rain i.L2temp_cat L2temp_cat##c.L2temp forest_area ib6.forest_border_cat forest_road forest_dist unemp) offset(logpop) difficult

estimates save Model_final.ster

// Prediction on whole dataset

use "~\TBE\TBE_pred_data.dta", clear

estimates use Model_final.ster

predict pred_final

outsheet id_municipality dekad pred_final using pred_final.csv , comma

// Margins

/* We decided to calculate margins for each variable by dividing it into equal intervals. */

// Example: obtaining margins for precipitation */

margins if L3rain>0 & L3rain<=10, at(L3rain=5) vsquish predict(ir)

margins if L3rain>10 & L3rain<=20, at(L3rain=15) vsquish predict(ir)

margins if L3rain>20 & L3rain<=30, at(L3rain=25) vsquish predict(ir)

margins if L3rain>30 & L3rain<=40, at(L3rain=35) vsquish predict(ir)

margins if L3rain>40 & L3rain<=50, at(L3rain=45) vsquish predict(ir)

margins if L3rain>50 & L3rain<=60, at(L3rain=55) vsquish predict(ir)

margins if L3rain>60 & L3rain<=70, at(L3rain=65) vsquish predict(ir)

margins if L3rain>70 & L3rain<=80, at(L3rain=75) vsquish predict(ir)

margins if L3rain>80 & L3rain<=90, at(L3rain=85) vsquish predict(ir)

margins if L3rain>90 & L3rain<=100, at(L3rain=95) vsquish predict(ir)
