# Supplementary material for: A Predictive Model Has Identified Tick-Borne Encephalitis High-Risk Areas in Regions Where No Cases Were Reported Previously, Poland, 1999–2012
Source: Int J Environ Res Public Health. 2018 Apr 4;15(4):677. doi: 10.3390/ijerph15040677 (PMC5923719; doi:10.3390/ijerph15040677)
Supplement: Supplementary file 1 [file ijerph-15-00677-s001.zip › ijerph-281680-supplementary/Supplementary file 3.docx]

**Supplementary materials for manuscript “A predictive model identified tick borne encephalitis high risk areas in regions were no cases were reported previously, Poland, 1999-2012”**

**Table S1. Comparison of the final model with all cases with a model excluding imported cases**

|  | **Model with all TBE cases (including non-residents)** | | **Model with local TBE cases only** | |
| --- | --- | --- | --- | --- |
| **Variable** | **Coefficient** | **Level of significance** | **Coefficient** | **Level of significance** |
| LOG-LINEAR PART |  |  |  |  |
| TBE cases (-1 dekad) | **0.215** | *** | **0.264** | *** |
| Sum of precipitation (-3 dekads) | **0.009** | *** | **0.008** | *** |
| Temperature index (if >0 degrees) | **2.071** | *** | **1.888** | *** |
| Mean temperature (-2 dekads) | -0.227 | NS | -0.253 | * |
| Interaction (temp. index * mean temp.) | 0.203 | NS | 0.224 | * |
| Forestation | **0.036** | *** | **0.031** | *** |
| Forest border density (ref: 6-9 m/ha) | - | - | - | - |
| 0-3 | 0.321 | NS | 0.283 | NS |
| 3-6 | **-0.505** | *** | **-0.471** | *** |
| 9-12 | **0.299** | ** | **0.366** | *** |
| > 12 | **0.736** | *** | **0.877** | *** |
| Forest road density | **-0.059** | ** | **-0.048** | ** |
| Average distance to forests | 0.139 | NS | 0.079 | NS |
| Unemployment | **0.047** | *** | **0.033** | *** |
| Constant in the model | **-11.197** | *** | **-10.842** | *** |
|  |  |  |  |  |
| LOGISTIC PART |  |  |  |  |
| TBE cases (-1 dekad) | **-0.839** | *** | -0.530 | NS |
| Sum of precipitation (-3 dekads) | -0.010 | * | -0.013 | * |
| Temperature index (if >0 degrees) | 1.600 | * | 1.754 | * |
| Mean temperature (-2 dekads) | **-0.493** | *** | **-0.475** | *** |
| Interaction (temp. index * mean temp.) | 0.219 | NS | 0.164 | NS |
| Forestation | **0.032** | *** | **0.023** | ** |
| Forest border density (ref: 6-9 m/ha) | - | - | - | - |
| 0-3 | 0.148 | NS | -0.008 | NS |
| 3-6 | -0.374 | NS | -0.187 | NS |
| 9-12 | 0.472 | * | 0.547 | * |
| > 12 | **1.074** | *** | **1.239** | *** |
| Forest road density | **-0.194** | *** | **-0.155** | ** |
| Average distance to forests | 0.568 | NS | 0.373 | NS |
| Unemployment | **0.072** | *** | **0.052** | ** |
| Constant in the model | 0.704 | NS | 1.345 | NS |
