# Supplementary material for: A Predictive Model Has Identified Tick-Borne Encephalitis High-Risk Areas in Regions Where No Cases Were Reported Previously, Poland, 1999–2012
Source: Int J Environ Res Public Health. 2018 Apr 4;15(4):677. doi: 10.3390/ijerph15040677 (PMC5923719; doi:10.3390/ijerph15040677)
Supplement: Supplementary file 1 [file ijerph-15-00677-s001.zip › ijerph-281680-supplementary/Supplementary file 4.docx]

**Supplementary materials for manuscript “A predictive model identified tick borne encephalitis high risk areas in regions were no cases were reported previously, Poland, 1999-2012”**

**Text S4. Results of the preliminary analysis of time series with number of cases reported from endemic municipalities**

We extracted cases from the 108 endemic municipalities. We constructed a single time series comprising a sum of cases in all these districts, by dekad.

*4.1. Periodogram*

The periodogram (Figure S5) has one dominating peak, which indicates existence of a cycle with frequency 0.028≈1/36 in natural frequencies (i.e. number of cycles per dekad). This corresponds to a cycle of the length of approximately 36 dekads, or 1 year.


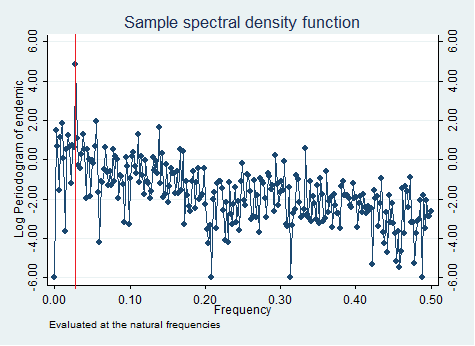


Figure S5. Periodogram, 108 endemic municipalities, 1999-2012.

*4.2. Partial autocorrelation plot*

The partial autocorrelation plot indicates autocorrelation in the time series of TBE cases (Figure S4.2). Especially, the first-order autocorrelation (lag 1) is high (0.8). Other significant autocorrelation coefficients are much smaller, and they are likely due to annual cycle (negative correlation with lags of approximately 0.5 year and positive with lags of approximately 1 year).


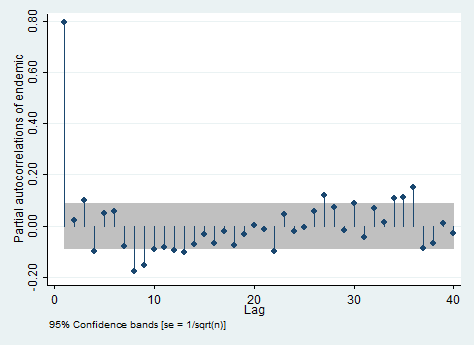


Figure S6. Partial autocorrelation plot, 108 endemic municipalities, 1999-2012.

*4.3. Dickey-Fuller test*

The Dickey – Fuller test is testing the null hypothesis of whether a unit root is present in an autoregressive model (Table S2). The significant result of this test indicates that the alternative hypothesis is true, i.e. our series is stationary.

|  | Test Statistic | 1% Critical Value | 5% Critical Value | 10% Critical Value |
| --- | --- | --- | --- | --- |
| Z(t) | -7.023 | -2.334 | -1.648 | -1.283 |
| p-value for Z(t) = 0.0000 | | | | |

Table S2. Augmented Dickey-Fuller test for unit root (n= 502), 108 endemic municipalities, 1999-2012.

*4.4. Longitudinal trend in the TBE times series*

The decomposition for seasonal, trend and random components showed no explicit increasing or decreasing trend in 10-year time series with number of cases from endemic municipalities (Figure S7).


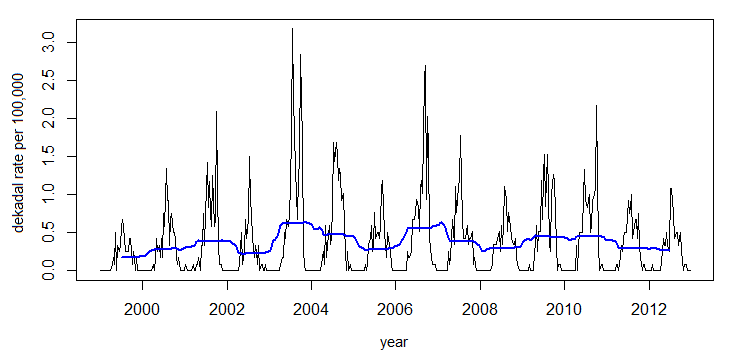


Figure S7. TBE cases time series (black) and trend component (blue), 108 endemic municipalities, 1999-2012.
