# Supplementary material for: A Predictive Model Has Identified Tick-Borne Encephalitis High-Risk Areas in Regions Where No Cases Were Reported Previously, Poland, 1999–2012
Source: Int J Environ Res Public Health. 2018 Apr 4;15(4):677. doi: 10.3390/ijerph15040677 (PMC5923719; doi:10.3390/ijerph15040677)
Supplement: Supplementary file 1 [file ijerph-15-00677-s001.zip › ijerph-281680-supplementary/Supplementary file 5.docx]

**Supplementary materials for manuscript “A predictive model identified tick borne encephalitis high risk areas in regions were no cases were reported previously, Poland, 1999-2012”**

**Table S3. Selection of lags for the meteorological measurements**

| lag precipitation |  | lag temperature | AIC |
| --- | --- | --- | --- |
| 1 |  | 1 | 15580,54 |
| 1 |  | 2 | 15568,93 |
| 1 |  | 3 | 15724,16 |
| 1 |  | 4 | 15905,84 |
| 2 |  | 1 | 15557,25 |
| 2 |  | 2 | 15537,26 |
| 2 |  | 3 | 15706,32 |
| 2 |  | 4 | 15897,06 |
| 3 |  | 1 | 15517,53 |
| **3** |  | **2** | **15498,81** |
| 3 |  | 3 | 15656,45 |
| 3 |  | 4 | 15866,46 |
| 4 |  | 1 | 15553,42 |
| 4 |  | 2 | 15552,15 |
| 4 |  | 3 | 15707,73 |
| 4 |  | 4 | 15905,70 |

Because there were significant associations between multiple dekadal measurements and TBE risk and autocorrelation between subsequent measurements, we decided to identify the lags for both variable, using the Akaike Information Criterion (AIC). The table summarizes all combination of lags for the association between mean temperature and sum of precipitation, and TBE onset.
