# Supplementary material for: A Predictive Model Has Identified Tick-Borne Encephalitis High-Risk Areas in Regions Where No Cases Were Reported Previously, Poland, 1999–2012
Source: Int J Environ Res Public Health. 2018 Apr 4;15(4):677. doi: 10.3390/ijerph15040677 (PMC5923719; doi:10.3390/ijerph15040677)
Supplement: Supplementary file 1 [file ijerph-15-00677-s001.zip › ijerph-281680-supplementary/Supplementary file 6.docx]

**Supplementary materials for manuscript “A predictive model identified tick borne encephalitis high risk areas in regions were no cases were reported previously, Poland, 1999-2012”**

**Text S6. Checking for the possibility of using independent variables as continuous in the log-linear model**

To check for the possibility to use independent variables as continuous in the log-linear model, we checked for their linear association with the TBE predicted rate using the below graphs. We constructed univariable models (the same predictor occurred both in logistic and log-linear part) including the tested predictor categorized into 5 categories of equal length. The natural logarithm (to account for the link function) of the predicted incidence for each level of the categorical version of the variable was plotted. The relation was approximately linear with exception of temperature and the length of forest border.


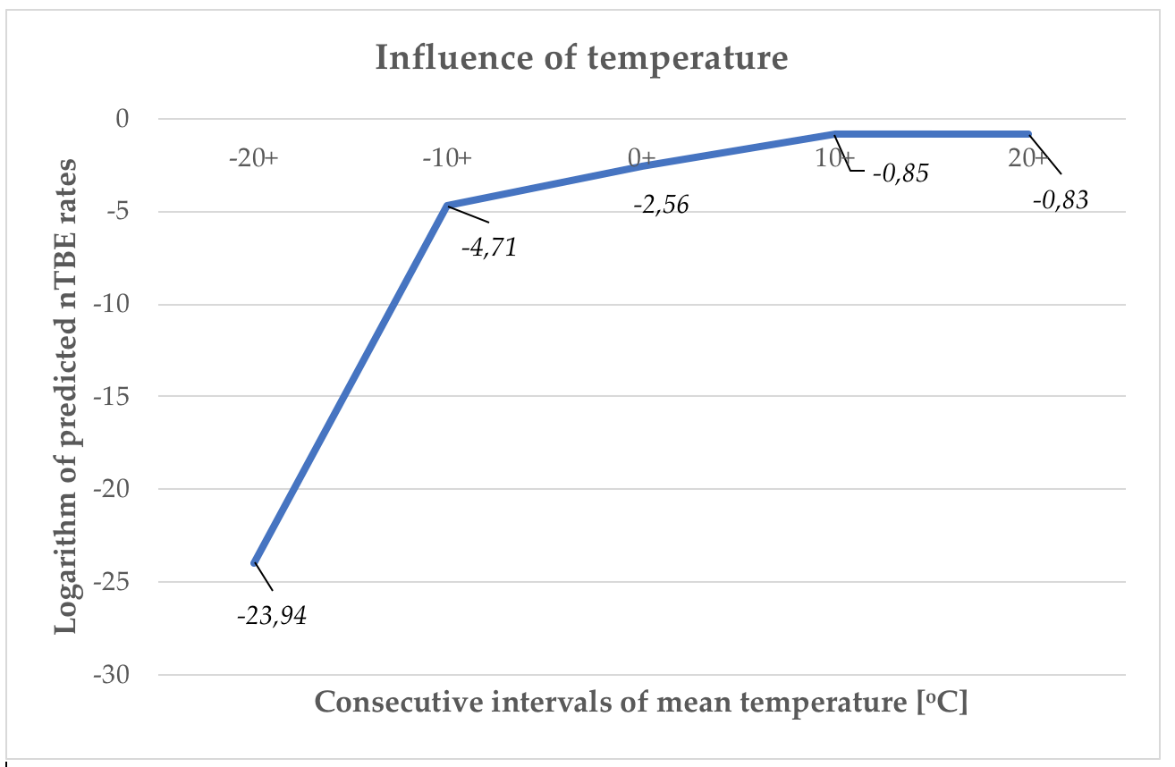


Figure S8. Univariable association between the mean temperature and

the logarithm of predicted TBE rate, 108 endemic municipalities, 1999-2012.


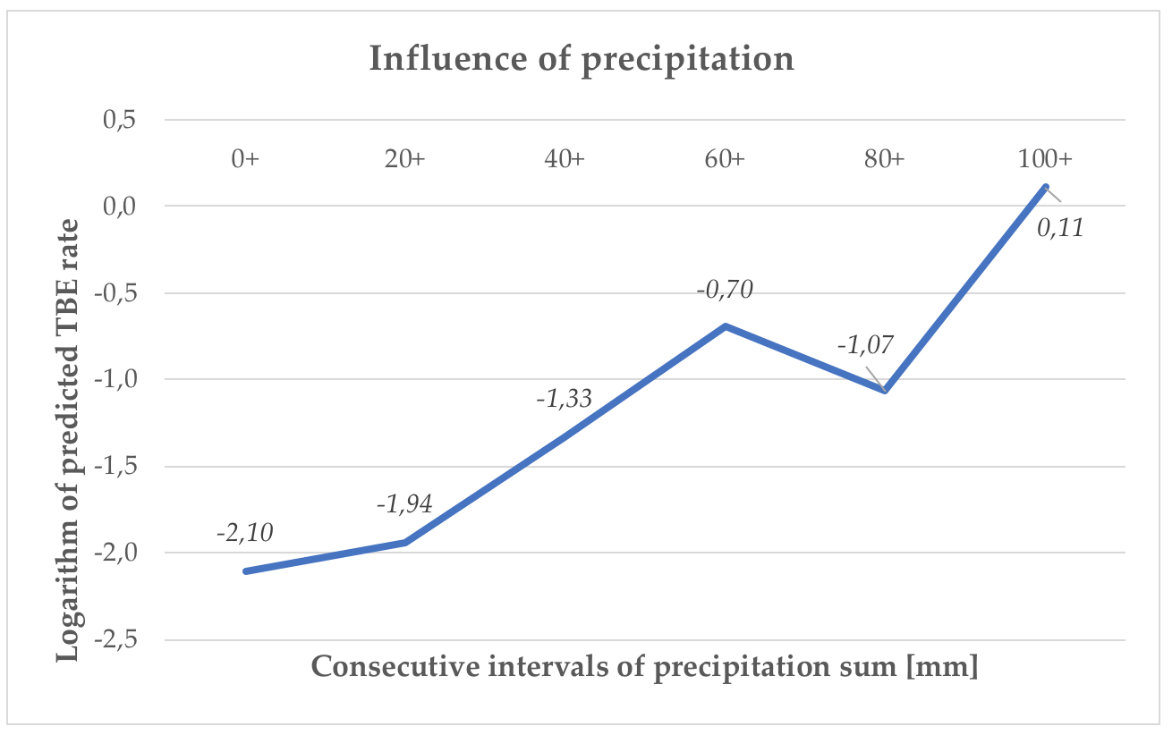


Figure S9. Univariable association between the sum of precipitation and

the logarithm of predicted TBE rate, 108 endemic municipalities, 1999-2012.


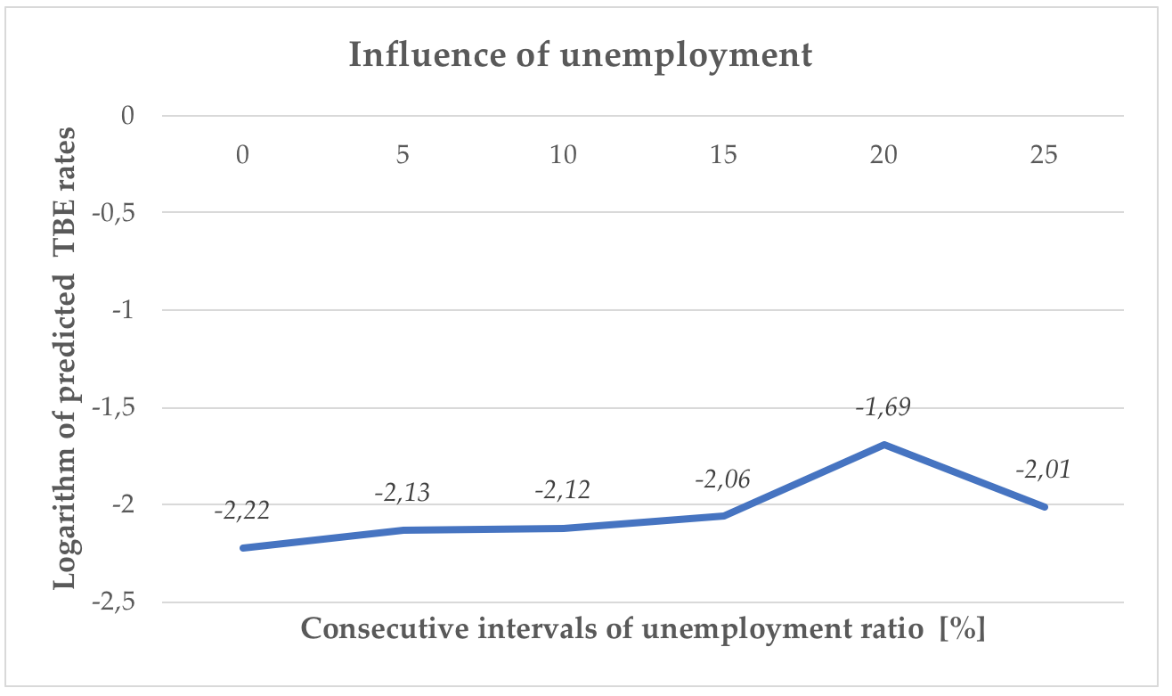


Figure S10. Univariable association between the unemployment ratio and

the logarithm of predicted TBE rate, 108 endemic municipalities, 1999-2012.


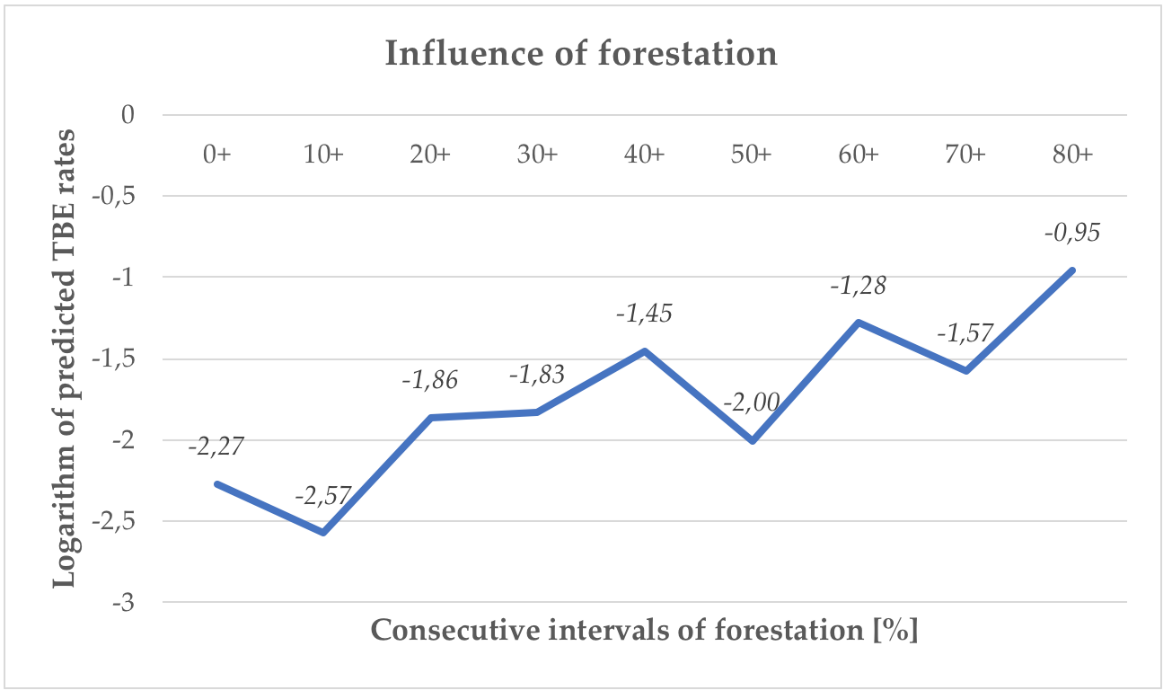


Figure S11. Univariable association between forestation and

the logarithm of predicted TBE rate, 108 endemic municipalities, 1999-2012.


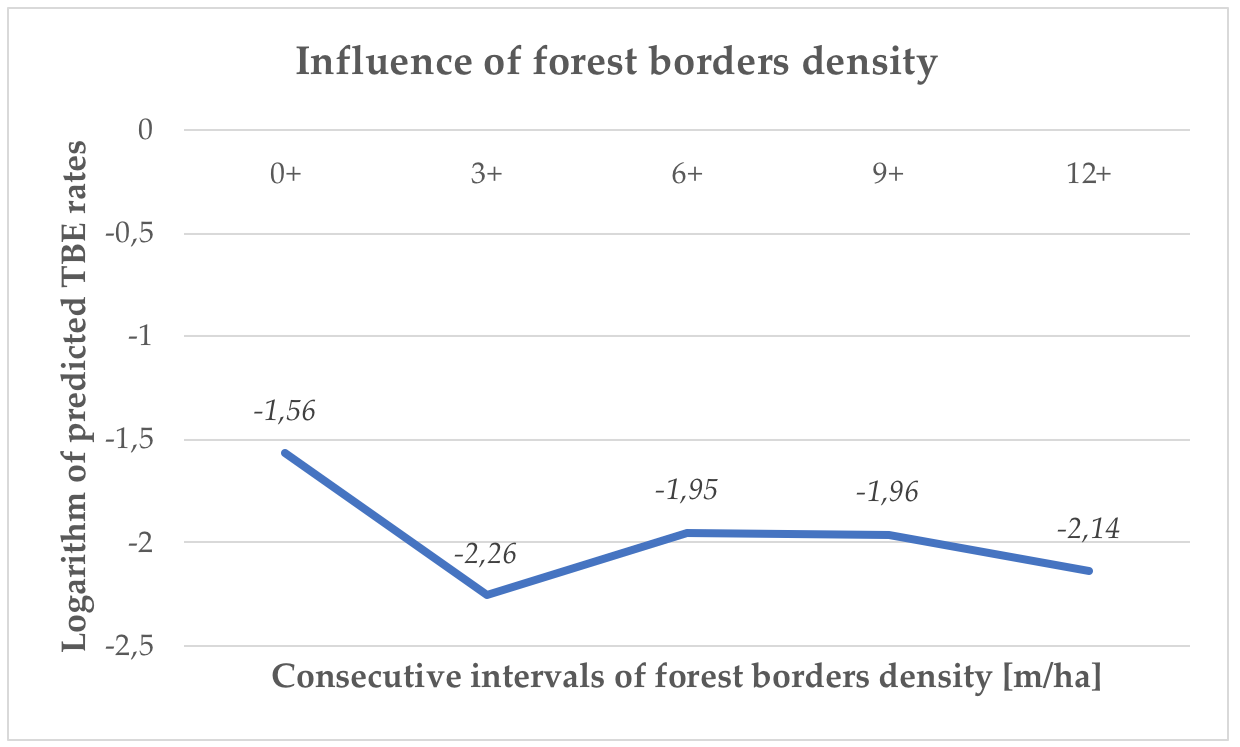


Figure S12. Univariable association between the forest borders density and

the logarithm of predicted TBE rate, 108 endemic municipalities, 1999-2012.


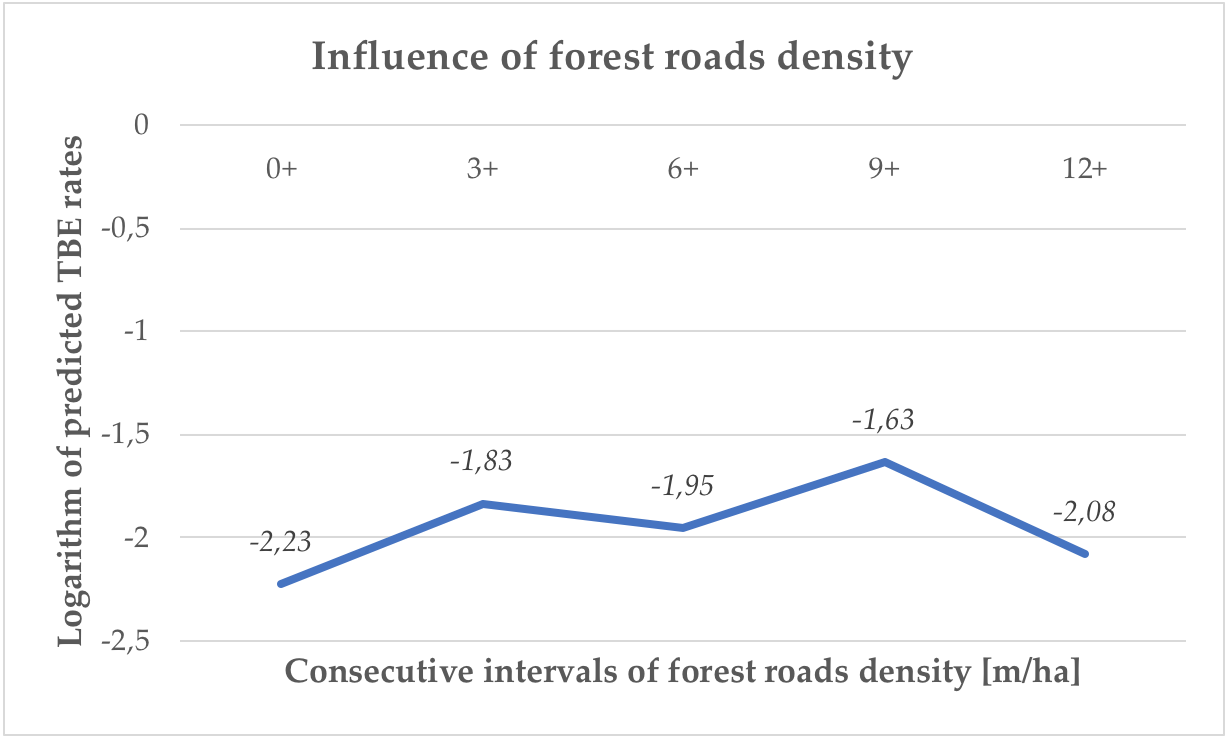


Figure S13. Univariable association between forest roads density and

the logarithm of predicted TBE rate, 108 endemic municipalities, 1999-2012.


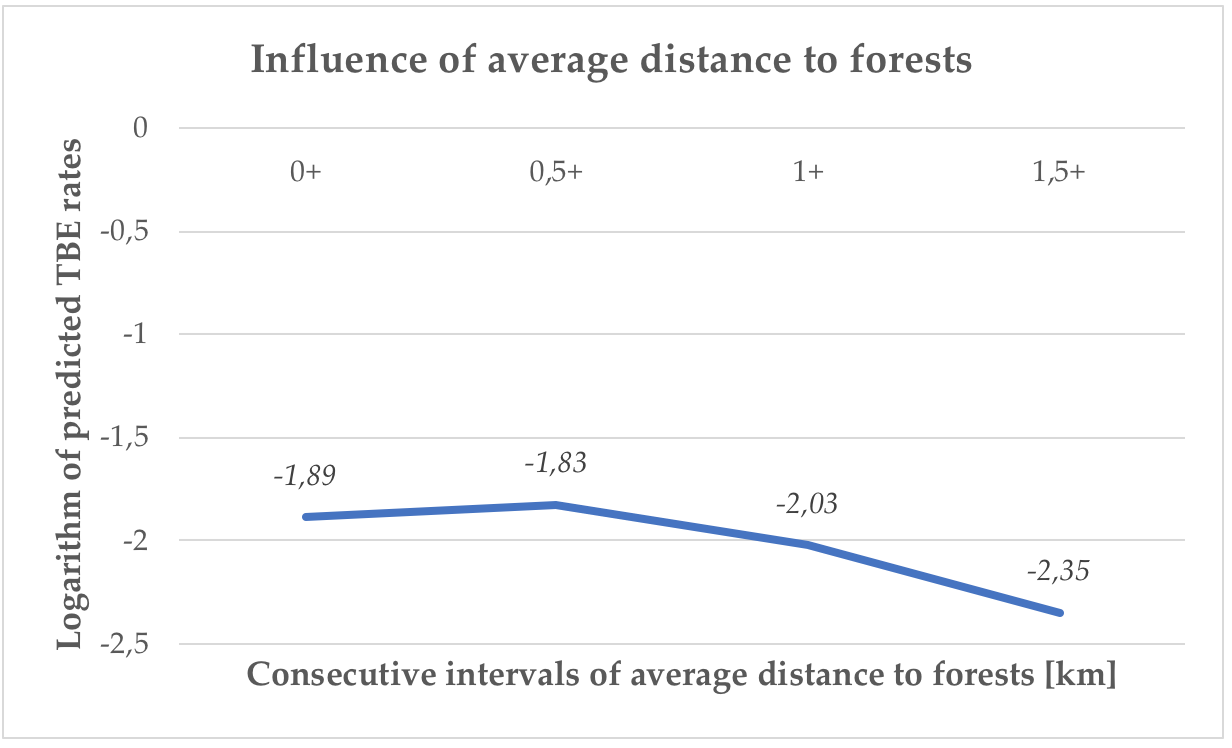


Figure S14. Univariable association between average distance to forests and

the logarithm of predicted TBE rate, 108 endemic municipalities, 1999-2012.
